# Natural Killer Cell Evasion Is Essential for Infection by Rhesus Cytomegalovirus

**DOI:** 10.1371/journal.ppat.1005868

**Published:** 2016-08-31

**Authors:** Elizabeth R. Sturgill, Daniel Malouli, Scott G. Hansen, Benjamin J. Burwitz, Seongkyung Seo, Christine L. Schneider, Jennie L. Womack, Marieke C. Verweij, Abigail B. Ventura, Amruta Bhusari, Krystal M. Jeffries, Alfred W. Legasse, Michael K. Axthelm, Amy W. Hudson, Jonah B. Sacha, Louis J. Picker, Klaus Früh

**Affiliations:** 1 Vaccine and Gene Therapy Institute, Oregon National Primate Research Center, Oregon Health and Science University, Beaverton, Oregon, United States of America; 2 Department of Life Sciences, Carroll University, Waukesha, Wisconsin, United States of America; 3 Department of Microbiology and Molecular Genetics, Medical College of Wisconsin, Milwaukee, Wisconsin, United States of America; University of California Berkeley, UNITED STATES

## Abstract

The natural killer cell receptor NKG2D activates NK cells by engaging one of several ligands (NKG2DLs) belonging to either the MIC or ULBP families. Human cytomegalovirus (HCMV) UL16 and UL142 counteract this activation by retaining NKG2DLs and US18 and US20 act via lysomal degradation but the importance of NK cell evasion for infection is unknown. Since NKG2DLs are highly conserved in rhesus macaques, we characterized how NKG2DL interception by rhesus cytomegalovirus (RhCMV) impacts infection *in vivo*. Interestingly, RhCMV lacks homologs of UL16 and UL142 but instead employs Rh159, the homolog of UL148, to prevent NKG2DL surface expression. Rh159 resides in the endoplasmic reticulum and retains several NKG2DLs whereas UL148 does not interfere with NKG2DL expression. Deletion of Rh159 releases human and rhesus MIC proteins, but not ULBPs, from retention while increasing NK cell stimulation by infected cells. Importantly, RhCMV lacking Rh159 cannot infect CMV-naïve animals unless CD8+ cells, including NK cells, are depleted. However, infection can be rescued by replacing Rh159 with HCMV UL16 suggesting that Rh159 and UL16 perform similar functions *in vivo*. We therefore conclude that cytomegaloviral interference with NK cell activation is essential to establish but not to maintain chronic infection.

## Author Summary

Natural killer (NK) cells are an important subset of the innate immune system that rapidly responds to cellular transformation and infection. The importance of NK cell control of viral infection is dramatically illustrated by our results revealing that cytomegalovirus (CMV) is unable to establish infections in healthy individuals unless NK cell responses are subverted. By studying infection of rhesus macaques with rhesus CMV, a highly representative animal model for human CMV, we identified a key viral factor that allows RhCMV to limit NK cell activation by preventing NK cell activating ligands from trafficking to the cell surface. Importantly, we observed that this avoidance of NK cell activation is essential to establish infection *in vivo* because RhCMV lacking the NK cell evasion factor was unable to infect animals unless NK cells were depleted. By unmasking such viral stealth strategies it might be possible to harness innate immunity to prevent viral infection, the primary goal of CMV vaccine development.

## Introduction

NK cells are a significant component of innate immunity against viruses and NK cell-deficient individuals are highly susceptible to herpesvirus infections [1]. Herpesviruses, particularly cytomegalovirus (CMV), encode proteins that either inhibit or activate NK cells and elegant studies with murine CMV (MCMV) revealed a complex relationship between NK cell stimulation and MCMV evasion during infection [2]. NK cell activation is controlled by inhibitory and activating receptors with inhibitory receptors, such as the KIR and CD94/NKG2 that recognize MHC-I, generally overriding positive signals [3]. Destruction of MHC-I by CMVs generates a “missing self” situation that reduces inhibitory signals [4].

A major activating receptor on NK cells is NKG2D, which is also expressed on γδ T cells, some CD4+ T cells, all αβ CD8+ T cell in humans, and activated and memory αβ CD8+ T cells in mice [5]. NKG2D interacts with multiple ligands: MHC-I related molecules (MICA and MICB) and the UL16-binding proteins (ULBP1-6) in humans, and the H60, MULT-1 and RAE-1 proteins in mice (reviewed in [5, 6]); all of which are induced upon cell stress including viral infection. Both human CMV (HCMV) and MCMV devote multiple gene products to prevent the surface expression of NKG2DL, presumably because the induction of any one can activate NKG2D [7]. In HCMV, UL16 retains ULBP1, 2, 6 and MICB in the endoplasmic reticulum (ER) [8–12] and MICB is additionally targeted by micro-RNA UL112 [13]. In addition, ULBP3 is retained by UL142 [14], whereas MICA is downregulated by UL142 [15], as well as US18 and US20 [16]. The fact that both virus and host devote multiple gene products to modulating NKG2D activation suggests an evolutionary arms race [17] that is exemplified by the observation that a recently evolved truncated allele of MICA (MICA*008) is counteracted by HCMV US9 [18].

The impact of NKG2DL interception by HCMV on primary infection and persistence, as well as on reinfection of seropositive individuals is not known. Studies in mice indicate that NKG2DL-inhibitory MCMV gene products are not required for infection but reduced viremia is observed in their absence [19]. Interestingly, replacing the NKG2DL-inhibitor m152 with RAE-1γ increased CD8+ T cell responses, both short and long term, despite viral attenuation [20]. Thus, increased NKG2D activation might improve the immunogenicity of CMV-based vectors while increasing safety.

CMV-based vectors are currently being developed for HIV/AIDS based on findings obtained with RhCMV-vectors in rhesus macaque (RM) models of HIV [21–23]. These studies revealed an unprecedented level of protection by RhCMV-based vaccines against Simian immunodeficiency virus (SIV) along with an unexpected ability of RhCMV to control T cell epitope targeting [24, 25]. The close evolutionary relationship between human and RM host also extends to the CMVs with the vast majority of HCMV genes conserved in RhCMV [26]. Interestingly however, while gene products involved in T cell control are largely conserved between RhCMV and HCMV [27, 28], the NKG2DL-inhibitory HCMV gene products, UL16 and UL142, are notably absent from RhCMV. In contrast, most NKG2DL are highly conserved in RM: MIC1 and MIC2 are closely related to MICA and MICB, respectively, whereas MIC3 is a chimera of MIC1 and MIC2 [29] and the RM genome encodes for ULBP1-4 are also highly conserved compared to humans [30] (S1 Fig). Given the conservation of the ligands but not of the viral NKG2DL-inhibitors, we examined whether RhCMV evolved unique NKG2DL-inhibitors. Using a panel of cell lines expressing human and rhesus NKG2DLs we demonstrate that RhCMV inhibits surface expression of all NKG2DLs tested and we identify Rh159, the homologue of HCMV UL148, as a major gene product responsible for retention of NKG2DLs. Similar to UL16, Rh159 prevents surface expression of multiple NKG2DLs. In contrast, UL148 did not retain NKG2DLs consistent with divergent evolution of protein function from a common ancestor. *In vitro*, deletion of Rh159 increased human and RM MIC protein expression and NK cell stimulation by RhCMV-infected cells. *In vivo*, RhCMV was unable to establish infection in either RhCMV sero-positive or sero-negative animals when Rh159 was deleted. However, primary infection occurred when the CD8+ cell population, that includes NK cells, was depleted. Moreover, infection of RM occurred when R159 was replaced with UL16 consistent with functional but not sequence homology between these two proteins. Our results suggest that NK cell evasion by NKG2DL downregulation is essential for infection by RhCMV and, given the conservation of host NKG2DL and the fact that UL16 can functionally replace Rh159, most likely by HCMV as well.

## Results

### RhCMV inhibits cell surface expression of NKG2DLs

**Fig 1:**
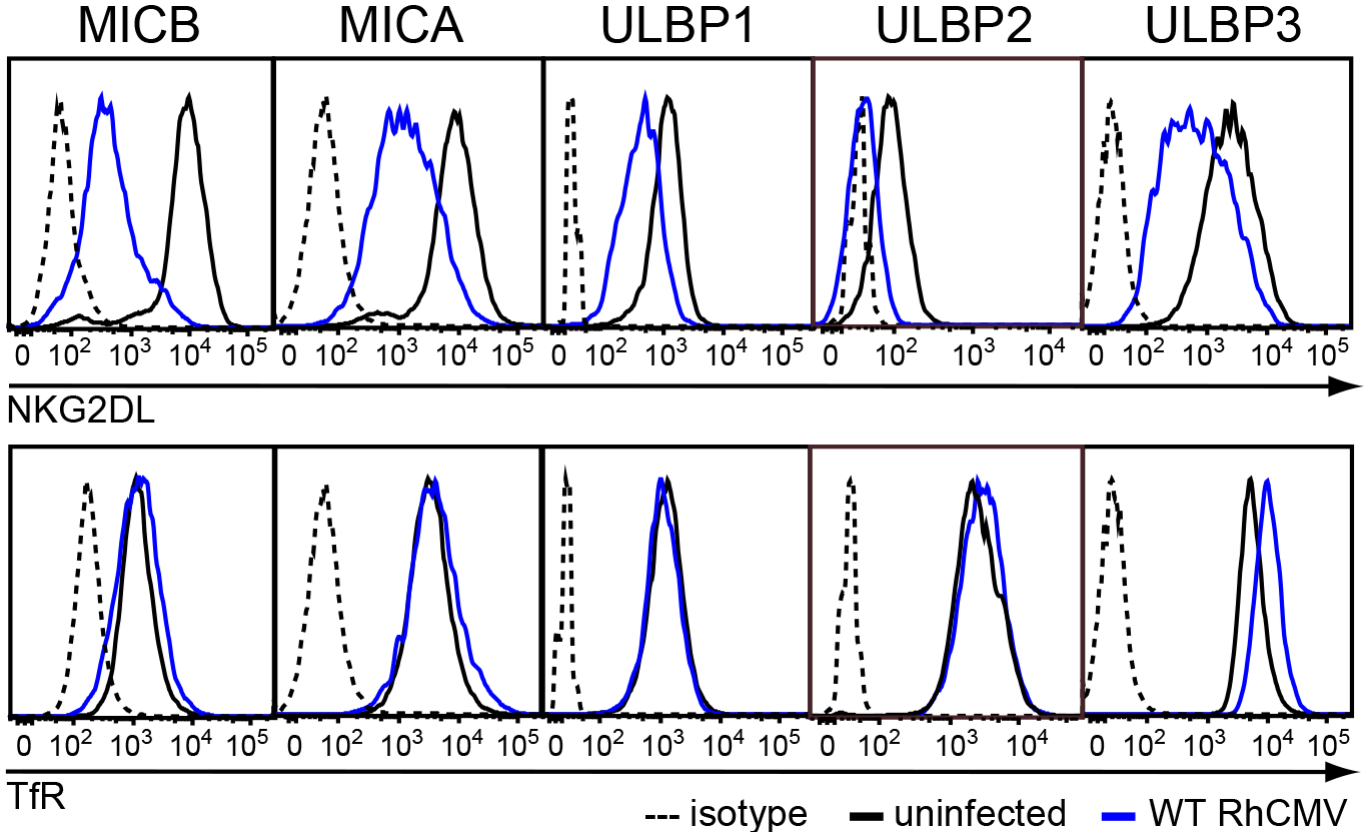
RhCMV reduces surface expression of NKG2DLs. U373-NKG2DL were infected with RhCMV (MOI = 3) (blue) or left uninfected (black). At 48 hpi surface levels of NKG2DL (upper panel) or TfR (lower panel) were determined by flow cytometry using specific antibodies or isotype control (dotted). Shown is NKG2DL or TfR cell surface expression on RhCMV-infected cells gated for IE2^+^. The results shown are representative of at least three independent experiments.

Since both MCMV and HCMV interfere with expression and intracellular transport of NKG2DLs, we hypothesized that RhCMV would similarly affect NKG2DL expression. Given the high degree of homology between human and RM NKG2DLs (S1 Fig), we took advantage of a panel of established U373 cell lines stably expressing human ULBP1-3, MICA, or MICB [U373-NKG2DL] [31]. RhCMV can productively infect U373 cells (S2 Fig). Therefore, we infected U373-NKG2DLs with RhCMV and determined whether RhCMV interferes with expression of these human NKG2DLs by monitoring the cell surface expression levels of each NKG2DL by flow cytometry (“RhCMV” refers to BAC-cloned RhCMV 68–1 [26] unless otherwise noted). Cell surface expression of NKG2DLs was assessed on infected cells by co-staining for IE2+ cells. At 48 hours post-infection (hpi), a substantial decrease in cell surface levels of each of the five human NKG2DLs was observed in RhCMV-infected cells (Fig 1). In contrast, RhCMV infection had a minimal impact on surface expression of transferrin receptor (TfR) suggesting that RhCMV specifically targets NKG2DLs. Since RhCMV interfered with expression of each of the human NKG2DLs and RM NKG2DLs (see below) examined, these data suggest that RhCMV targets the full panel of NKG2D-activating ligands presumably to prevent NKG2D-dependent activation of NK cells.

### MICB is retained in the ER/cis-Golgi and associates with Rh159 in RhCMV-infected cells

**Fig 2:**
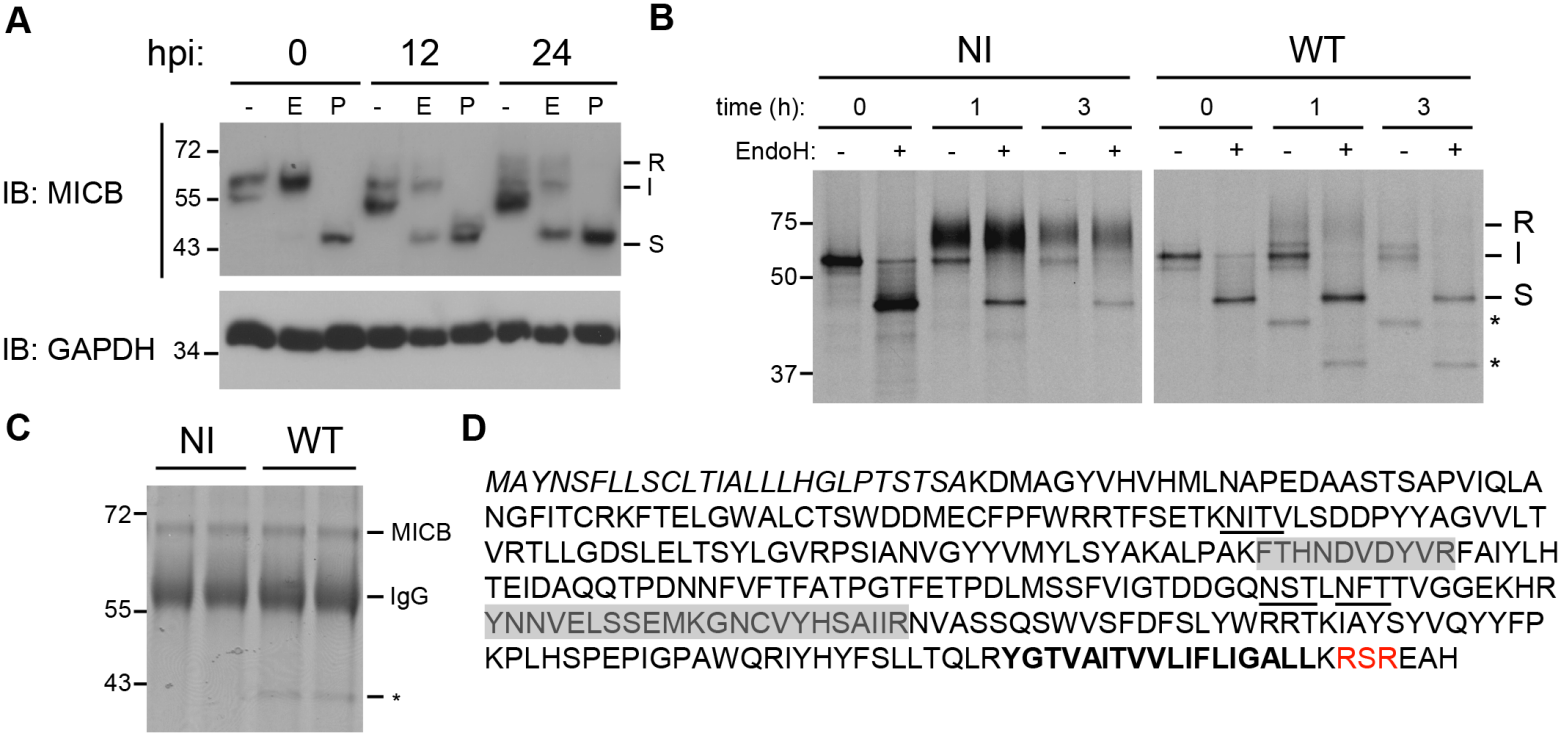
MICB is retained in the ER and associates with Rh159 in RhCMV infected cells. **A)** Immature MICB is enriched in RhCMV-infected cells. U373-MICB cells were infected with RhCMV (MOI = 3) for 12 or 24 h or left uninfected prior to lysis in 1% NP40. Cell lysates were treated with Endoglycosidase H (E), PNGase F (P), or digestion buffer alone (-), separated by SDS-PAGE and immunoblotted with monoclonal antibodies (mAbs) to MICB or GAPDH. Mature glycosylated MICB (67kDa) is EndoH resistant (R), whereas immature MICB (I) remains EndoH sensitive with an apparent MW of 43kDa upon EndoH treatment (S) indicative of ER retention. **B)** RhCMV inhibits intracellular transport of MICB. U373-MICBs were infected with RhCMV (WT) (MOI = 3) for 24 h (visualization by light microscopy confirmed 100% cytopathic effect (CPE)) or left uninfected (NI) followed by metabolically labeling with [35S]cysteine and [35S]methionine for 30 min. The label was then chased for the indicated times (h), cells were lysed and MICB was immunoprecipitated from cell lysates using a MICB specific antibody. Precipitates were either digested with EndoH (+) or mock treated (-) followed by SDS-PAGE and autoradiography. Stars (*) indicate an EndoH-sensitive protein co-precipitating with MICB in infected cells. **C-D)** Isolation and identification of Rh159 co-immunoprecipitating with MICB. U373-MICB cells were infected with RhCMV (WT) or left non-infected (NI) as above and cells were lysed at 48 hpi. MICB was immunoprecipitated with anti–MICB mAb, the immunoprecipitates were separated by SDS-PAGE, and proteins visualized by Coomassie Blue staining. The IgG heavy chain (55kDa) is indicated. The 43kDa protein (*) was excised from the gel and digested with trypsin. **D)** Mass-spectrometric analysis by LC-MS/MS identified tryptic peptides corresponding to Rh159 (gray shaded boxes). The predicted amino acid sequence of Rh159 contains a signal sequence (italics), N-linked glycosylation sites (underlined), a C-terminal transmembrane anchor (bold), and an RXR ER retrieval motif (red). The results shown in A and B are representative of two or more independent experiments.

We hypothesized that RhCMV targeted NKG2DL by a post-translational mechanism since transcription of these genes is controlled by heterologous transcriptional and translational control elements in U373-NKG2DL cells [31]. Therefore, we monitored glycosylation of MICB over 24 h of infection by collecting cells at various hpi and digesting cell lysates with Endoglycosidase H (EndoH) or Peptide N-Glycosidase F (PNGaseF) prior to electrophoretic separation and immunoblot with MICB-specific antibodies. Over time, increasing amounts of EndoH-sensitive compared to EndoH-resistant MICB protein accumulated in RhCMV-infected cells (Fig 2A). Since EndoH specifically removes high mannose oligosaccharides generated in the ER, EndoH sensitivity suggests that RhCMV retained MICB in the ER/cis-Golgi. The decrease of EndoH-resistant MICB over time mirrors the turnover of MICB seen in uninfected cells and thus was likely due to natural turnover [32]. To determine whether RhCMV specifically targeted newly synthesized MICB, we immunoprecipitated MICB from metabolically pulse/chase labeled U373-MICB cells at 24 hpi. In uninfected cells, the majority of MICB was EndoH resistant at 1 h post-chase (Fig 2B, left panel). In contrast, MICB did not attain EndoH-resistance in RhCMV-infected cells even at 3 h (Fig 2B, right panel), which was consistent with RhCMV preventing MICB maturation. Interestingly, an additional protein species (indicated by *) was observed in MICB-immunoprecipitations in RhCMV-infected, but not in uninfected samples at the 1 h and 3 h time point (Fig 2B). We hypothesized that the co-precipitating protein, which seemed to be ER-resident since it remained EndoH-sensitive throughout the chase period, was of viral origin. To identify this glycoprotein, we performed preparative MICB-immunoprecipitations from RhCMV-infected U373-MICB cells for analysis by mass-spectrometry (MS). When the immunoprecipitated proteins were separated by SDS-PAGE and visualized by Coomassie-staining, only MICB was observed in samples from uninfected cells whereas approximately equal amounts of MICB and the unknown ~40kDa species were co-precipitated by MICB-specific antibodies from RhCMV-infected cells (Fig 2C). The ~40kDa protein was excised from the gel, digested with trypsin and analyzed by liquid-chromatography tandem MS (LC-MS/MS). Multiple peptide-species were identified that corresponded in mass to predicted peptides of Rh159 (Fig 2D), a putative RhCMV glycoprotein with a predicted MW of 36.8 kDa (34.1 kDa without the putative signal peptide) that is similar to the observed MW of the (de-glycosylated) viral protein co-immunoprecipitating with MICB (Fig 2B). Rh159 is predicted to contain two N-linked glycosylation sites and to display a type1b transmembrane protein topology with a cleavable signal sequence. A very short cytoplasmic tail of seven amino acids encompasses a putative RXR ER retrieval motif (Fig 2D). Rh159 was thus a strong candidate for an ER-resident viral glycoprotein that associates with and retains newly synthesized NKG2DL by hijacking the cellular ER-retrieval mechanism.

### Rh159 retains MICB in the ER/cis-Golgi

**Fig 3:**
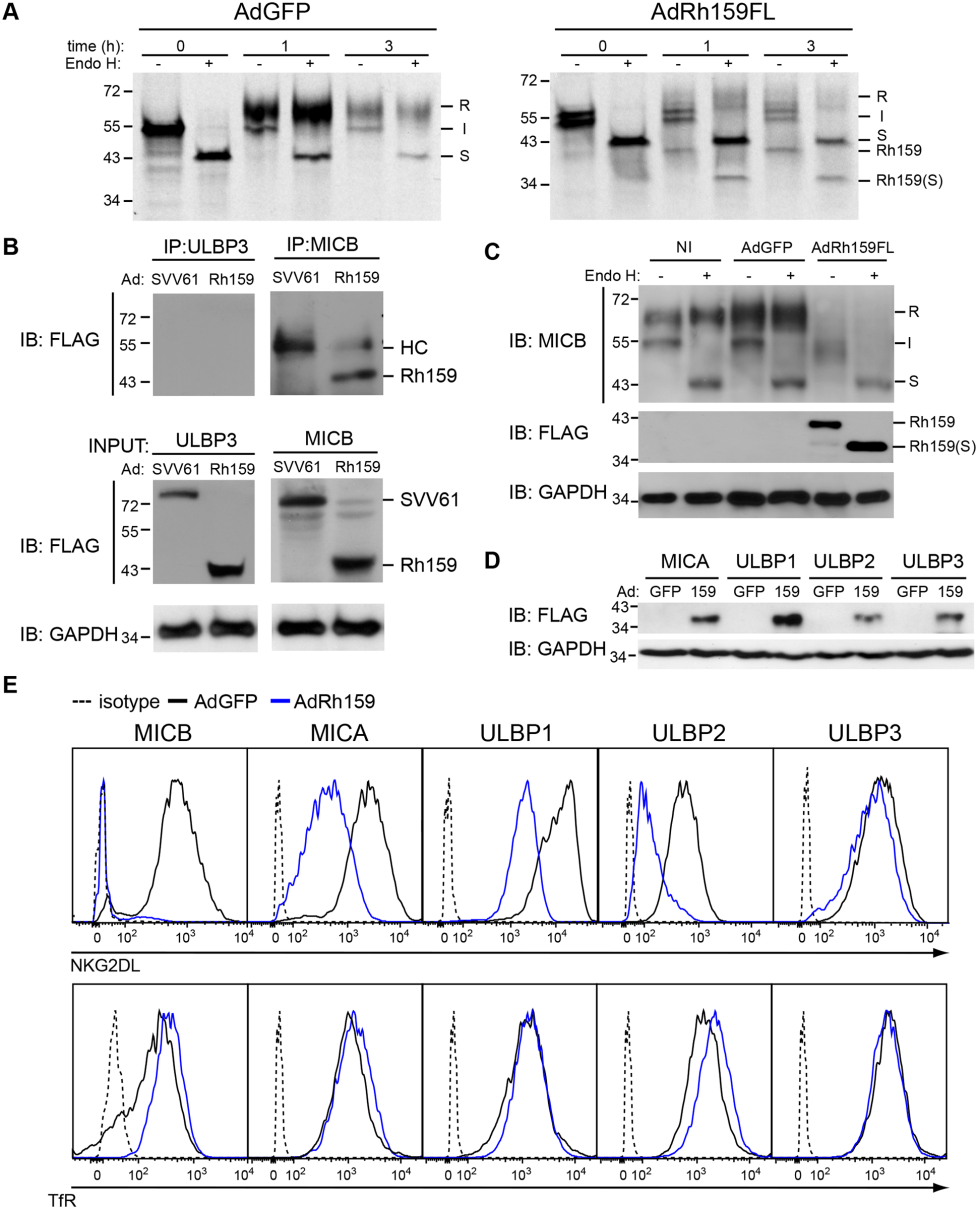
Rh159 interferes with intracellular transport of NKG2DL. **A)** Association with Rh159 prevents intracellular transport of MICB. U373-MICB cells were transduced with adenovectors (MOI = 80) expressing either GFP (AdGFP) or FLAG-tagged Rh159 (AdRh159FL) under control of tetracycline-dependent transactivator provided by co-transduced AdtTA (MOI = 20). At 24 hpi cells were metabolically labeled for 30 min with [35S]cysteine + [35S]methionine. Upon chasing the label for the indicated times (h), cells were lysed and MICB was immunoprecipitated with anti–MICB mAb. Precipitates were either digested with EndoH (+) or mock treated (-) followed by SDS-PAGE and autoradiography. (S) indicates EndoH-deglycosylated proteins. **B)** Rh159 co-immunoprecipitates with MICB. U373-ULBP3 (ULBP3, left panel) or U373-MICB (MICB, right panel) cells were lysed at 48 h post-transduction with AdRh159FL (Rh159) or an adenovector expressing FLAG-tagged SVV ORF 61 (SVV61) used as a negative control. MICB and ULBP3 were immunoprecipitated with anti–MICB and anti-ULBP3 mouse and goat mAbs, respectively, then immunoblotted with mouse anti-FLAG mAb. The mouse IgG heavy chain (55kDa) is indicated (HC). Input lanes were loaded with 10% total lysate used in immunoprecipitation and immunoblotted with mAbs for FLAG and GAPDH. The results shown are representative of two independent experiments. **C)** Rh159 reduces steady state levels of MICB. U373-MICB cells were lysed at 48 h post-transduction with the indicated Ad-vectors. Lysates were digested with EndoH (+) or mock treated (-) then immunoblotted with mAbs for MICB, FLAG or GAPDH. Note that both MICB and Rh159 are EndoH sensitive consistent with ER localization. The results shown are representative of two independent experiments. **D-E)** Rh159 reduces surface expression of MICA, MICB, ULBP1 and ULBP2 but not ULBP3. U373-NKG2DL cells were transduced with AdRh159FL or AdGFP as in A) but for 48 h. Cells were then lysed and immunoblotted with mAbs for FLAG and GAPDH (**D**), or stained with antibodies specific for the indicated proteins, or isotype control (dotted) and analyzed by flow cytometry. The results shown are representative of three or more independent experiments.

To determine whether Rh159 was sufficient to retain MICB, we inserted codon-optimized Rh159 (including a C-terminal FLAG-epitope tag) into a replication-deficient adenovector under control of the tetracycline-regulated promoter. U373-MICB cells were co-transduced with the corresponding construct (AdRh159FL) and an adenovector expressing the tetracycline-regulated transactivator (AdtTa). For control we used an adenovector expressing GFP (AdGFP). At 24 hpi, MICB was immunoprecipitated from Ad-transduced U373-MICB cells at 0, 1, and 3 h post- chase. In U373-MICB cells transduced with AdGFP, the majority of newly synthesized MICB attained EndoH resistance within 1h post-chase (Fig 3A). In contrast, the majority of MICB remained EndoH-sensitive throughout the chase period in AdRh159FL-transduced cells (Fig 3A). We further observed that MICB-specific antibodies co-immunoprecipitated an EndoH-sensitive protein of ~40KDa from AdRh159FL-transduced cells but not from AdGFP-transduced cells (Fig 3A). The ~40kDa band was identified as Rh159 by immunoblot with anti-FLAG antibodies (Fig 3B) whereas the band was not observed in U373-ULBP3 cells transduced with AdRh159FL or when an adenovector expressing C-terminal FLAG tagged Simian varicella virus (SVV) open reading frame (ORF) 61 was used [33]. Interestingly, steady state protein levels of MICB were strongly reduced and were mostly EndoH sensitive at 48 hpi with AdRh159FL, suggesting that prolonged retention ultimately leads to degradation of MICB (Fig 3C). Taken together, these data demonstrate that Rh159 associates with and retains MICB in the ER.

To determine whether Rh159 also targets other NKG2DLs, we co-transduced the U373-NKG2DL panel with AdtTA and AdRh159FL or AdGFP. Expression of Rh159 was confirmed by immunoblot (Fig 3D) and NKG2DL-surface expression was monitored by flow cytometry (Fig 3E). Mean fluorescence intensity (MFI) of MICB was reduced by more than 2 orders of magnitude upon transduction with AdRh159FL compared to AdGFP whereas cell surface levels of TfR were not affected (Fig 3E). Similarly, Rh159-expression reduced surface levels of MICA, ULBP1, and ULBP2 whereas expression of ULBP3 was not affected (Fig 3E). Thus, Rh159 impairs the surface expression of most, but not all, NKG2DLs.

### HCMV UL148 does not downregulate NKG2DL

**Fig 4:**
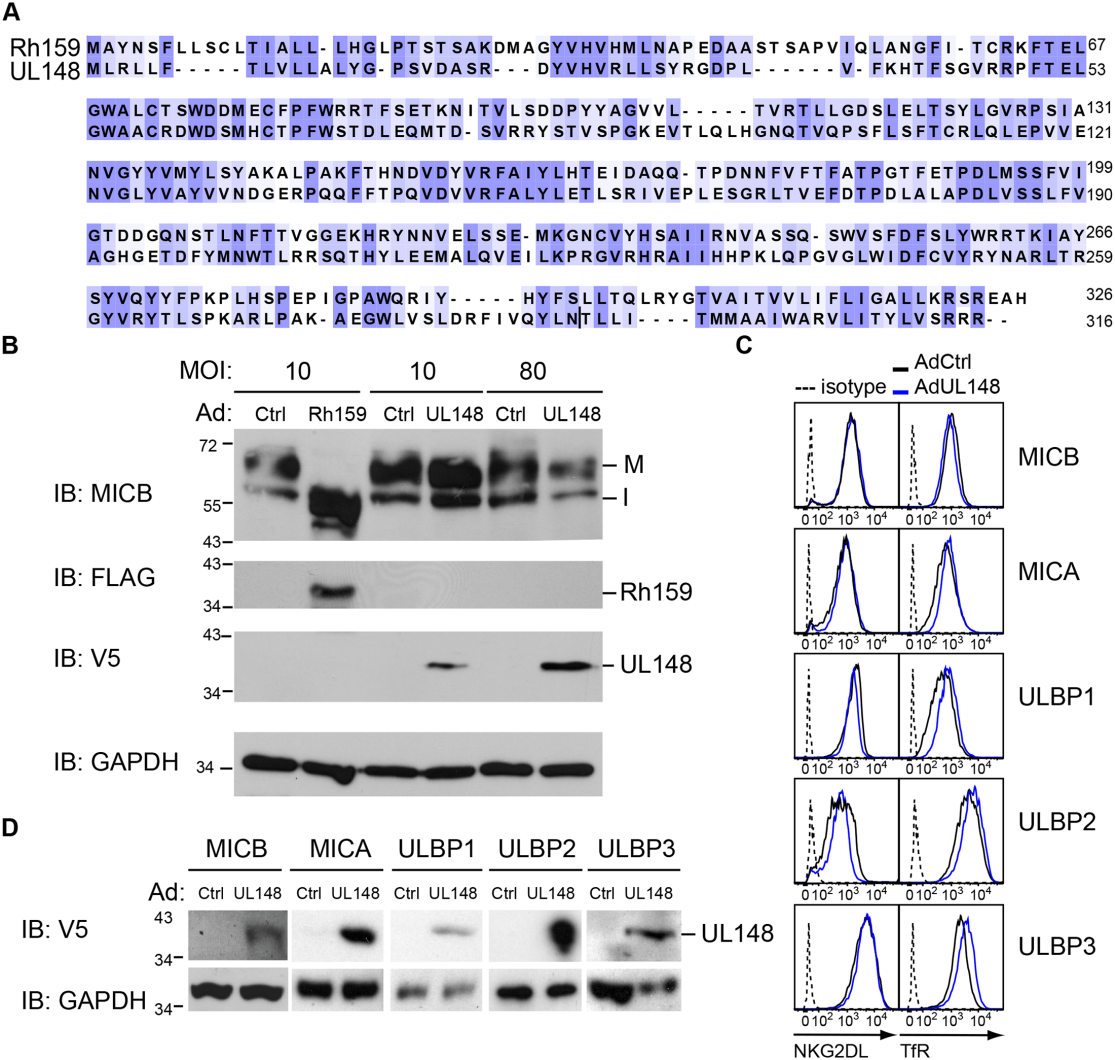
Cell surface expression of NKG2DLs is not affected by HCMV UL148. **A)** Alignment of Rh159 and UL148. Intensity of purple shading indicates level of sequence conservation (Jalview 2 [55]). **B**) MICB maturation is not affected by UL148. U373-MICB cells were transduced with AdCtrl (empty vector) (Ctrl), or AdUL148 containing a C-terminal V5 tag (UL148) at an MOI of 10 or 80, or AdRh159FL (Rh159) (MOI = 10) together with AdtTA (MOI = 2.5) for 48 h. UL148 and Rh159 expression was verified by immunoblot using anti-V5 antibody or anti-FLAG antibody, respectively, with antibodies to GAPDH providing a loading control. The mature MICB protein of 67 kDA is designated M whereas I refers to immature MICB retained in the ER by Rh159. Results shown are representative of two experiments. **C)** UL148 does not affect cell surface expression of NKG2DL. U373-NKG2DL cells were transduced with AdUL148 (blue) or AdCtrl (black) (MOI = 80) and, at 48 h, cells were harvested and analyzed for NKG2DL and TfR surface expression compared to isotype controls (dotted) by flow-cytometry. **D)** UL148 expression in C) was verified by immunoblot in each of the samples using anti-V5 antibody. GAPDH served as a loading control.

RhCMV Rh159 shares 30% sequence identity with HCMV UL148 [34], comparable to other RhCMV proteins demonstrated to be functional HCMV protein homologues [26, 27] (Fig 4A). To determine whether HCMV UL148 would target NKG2DL expression, we transduced each of the U373-NKG2DLs with a previously described adenovector expressing UL148 including a C-terminal V5-epitope tag [16]. However, in contrast to Rh159, MICB maturation was not affected by UL148 even at high MOI (Fig 4B) and MICB surface expression remained unchanged after transduction with Ad148 (Fig 4C). Similarly, cell surface levels of MICA, ULBP1, ULBP2 and ULBP3 were not affected by UL148 (Fig 4C). Since UL148 expression was verified by immunoblot (Fig 4D), we conclude that despite sequence and positional homology, UL148 and Rh159 diverge in their ability to target NKG2DL. While we cannot rule out that UL148 targets a NKG2DL not tested here, previous reports showed increased NKG2DL surface expression upon infection with HCMV lacking UL16 but containing functional UL148 [16]. In addition, no decrease in NK cytotoxicity was found when cells transduced with Ad148 were co-incubated with human NK cells when compared to control [35] consistent with our conclusion that HCMV UL148 is not an NK cell evasion factor despite common ancestry with Rh159.

### Deletion of Rh159 restores intracellular maturation and cell surface expression of human MIC proteins in RhCMV-infected U373 cells

**Fig 5:**
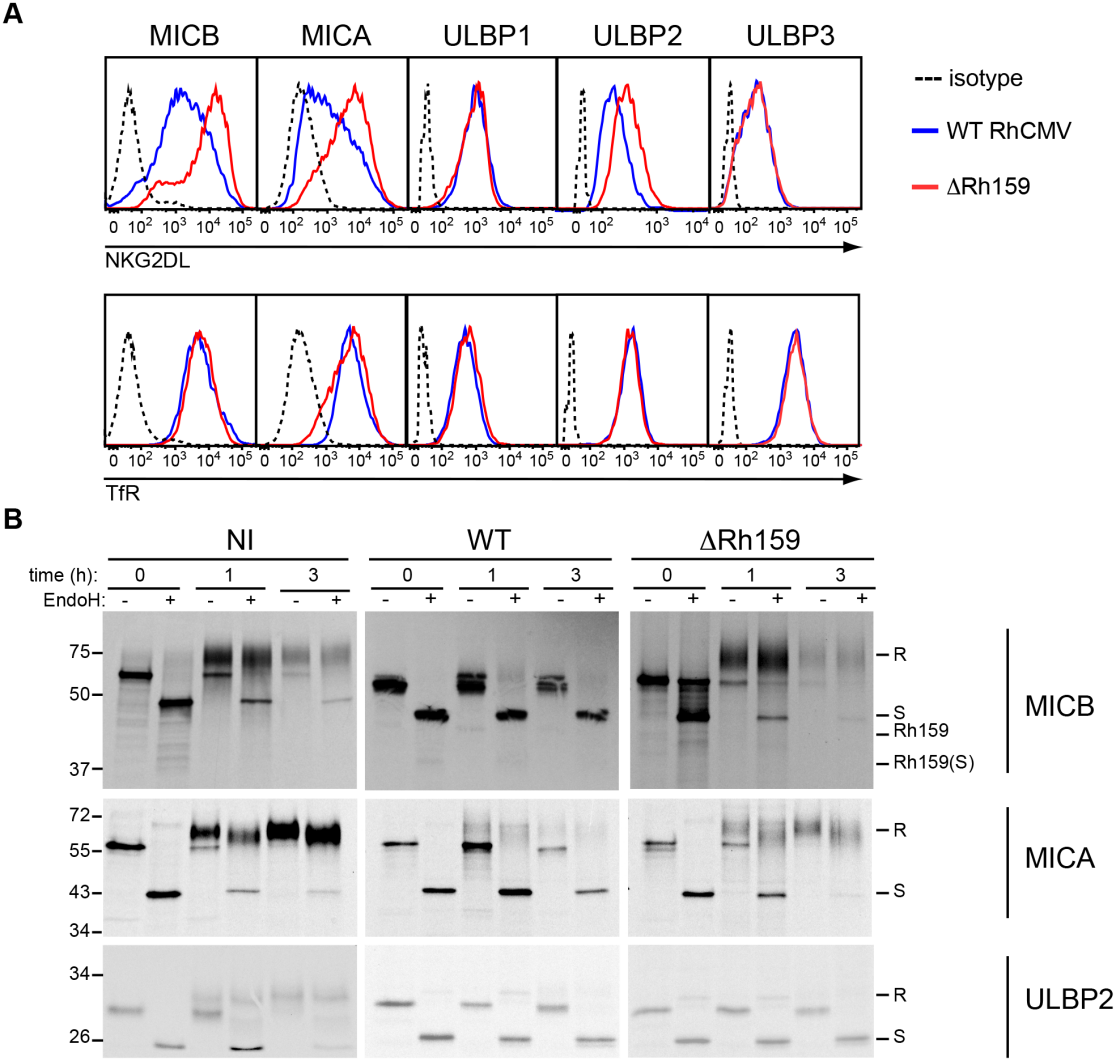
Deletion of Rh159 rescues intracellular transport and surface expression of MICA and MICB upon RhCMV infection. **A)** Comparison of NKG2DL surface expression upon infection with RhCMV or ΔRh159. U373-NKG2DL cells were infected with RhCMV (blue) or ΔRh159 (red) (MOI = 3) for 48 h. Cell surface levels of NKG2DL or TfR were determined by flow cytometry, using specific antibodies and compared to isotype control (dotted). Depicted is NKG2DL or TfR surface expression on infected cells gated for RhCMV IE2^+^ expression. The results shown are representative of three or more independent experiments. **B)** Biosynthesis and maturation of NKG2DL in uninfected U373-NKG2DL cells or upon infection with RhCMV or ΔRh159. U373-NKG2DL cells were uninfected (NI), infected with RhCMV (WT) or ΔRh159 (MOI = 3) for 24 h, verified by light microscopy as having 100% CPE, then metabolically labeled with [35S]cysteine and [35S]methionine for 30 min prior to chasing the label for the indicated times. The indicated NKG2DLs were immunoprecipitated from cell lysates with specific mAbs. Immunoprecipitates were split and digested with EndoH (+) or mock treated (-) then analyzed by SDS-PAGE and autoradiography.

To further demonstrate that Rh159 is responsible for ER-retention of MICB, we created a Rh159 knock out virus (ΔRh159) by replacing Rh159 with SIVgag using BAC mutagenesis (S2A and S2B Fig). Replacement of Rh159 with SIVgag had only a modest impact on viral growth in tissue culture (S2C Fig) and the viral genome was stable upon multiple passages as shown by full genome sequencing (S2D Fig). To determine the contribution of Rh159 to NKG2DL downregulation by RhCMV we compared NKG2DL surface levels in U373-NKG2DL cells infected with RhCMV or ΔRh159. Interestingly, both MICA and MICB surface levels were significantly higher in cells infected with ΔRh159 compared to RhCMV (Fig 5A). In contrast, only a minor increase of ULBP2 surface levels was observed in the absence of Rh159 and, as expected, ULBP3 levels were not increased (Fig 5A). Although exogenous expression of Rh159 reduced ULBP1 surface levels (Fig 3E), infection with RhCMV in the absence of Rh159 did not lead to increased ULBP1 surface levels compared to RhCMV (Fig 5A). Since MICA, MICB, and ULBP2 surface levels were significantly higher in cells infected with ΔRh159 compared to RhCMV, we wanted to specifically address the impact of Rh159 on the maturation of these NKG2DLs when newly synthesized during infection. Therefore, we examined NKG2DL maturation by pulse-chase labeling and immunoprecipitation upon infection with ΔRh159. In contrast to RhCMV-infected U373-MICB cells, MICB acquired EndoH resistance upon infection with ΔRh159 at the same rate as observed for uninfected cells (Fig 5B) suggesting that intracellular transport of MICB was no longer inhibited. Also note that the co-precipitating viral protein seen in Figs 2B and 5B is absent in ΔRh159-infected cells. Similarly, MICA maturation was unimpeded upon infection with ΔRh159 (Fig 5B) consistent with the increased MICA surface expression compared to RhCMV (Fig 5A). However, ULBP2 was still retained by ΔRh159 despite the fact that AdRh159FL downregulated these proteins (Fig 3E). The slight increase of ULBP2 in ΔRh159-infected cells (Fig 5A) was thus not due to lack of ULBP2 retention in the absence of Rh159. Taken together, these results suggest that RhCMV encodes additional proteins targeting ULBPs. In contrast, Rh159 seems to be predominantly, if not solely, responsible for the retention of MICA and MICB by RhCMV.

### Deletion of Rh159 increases NK cell stimulation by RhCMV-infected cells

**Fig 6:**
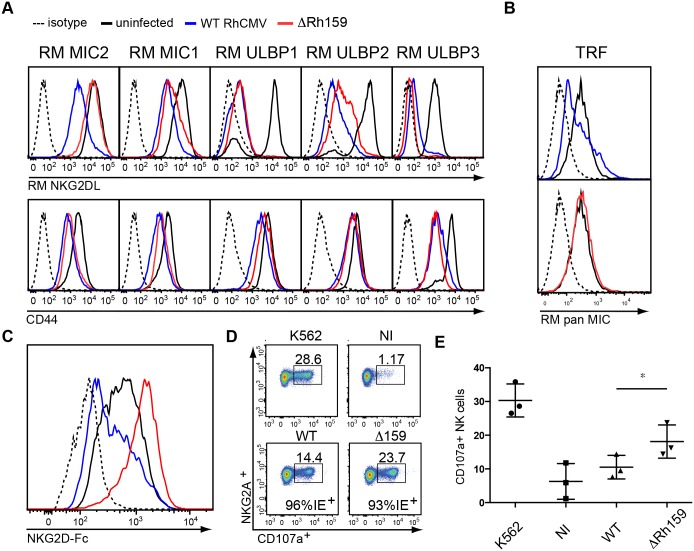
Deletion of Rh159 increases RM MIC2 expression and NK cell stimulation by RhCMV-infected cells *in vitro*. **A**) Surface expression of RM NKG2DLs upon infection with RhCMV or ΔRh159. N-terminally HA-tagged RM NKG2DLs (S1 Fig) were stably expressed in telomerized RM fibroblasts (TRF) upon lentivector transduction. TRF-RM NKG2DL were infected with RhCMV (blue), ΔRh159 (red) (MOI = 3) or left uninfected (black). At 48 hpi, surface levels of RM NKG2DLs (upper panel) or CD44 (lower panel) were determined by flow cytometry using anti-HA or anti-CD44 antibodies (respectively) or isotype control (dotted line). Shown is NKG2DL or CD44 cell surface expression on RhCMV-infected cells gated for IE2^+^. The results shown are representative of at least three independent experiments. **B**) Upregulation of endogenous RM MIC proteins by ΔRh159 compared to RhCMV. TRFs were infected with RhCMV, ΔRh159 (MOI = 3) or uninfected. At 48h, cells surface expression of RM MIC was determined by flow cytometry using anti-RM pan MIC antibody [46](kindly provided by T. Spies). Shown is RM pan MIC cell surface expression on RhCMV-infected cells gated for IE2^+^. **C)** Increased NKG2D-binding to RM fibroblasts infected with ΔRh159 compared to RhCMV. TRFs were infected with RhCMV (blue), ΔRh159 (red) (MOI = 3) or left uninfected (black) for 48 h. Cell surface levels of NKG2DL were determined by flow cytometry using a recombinant human NKG2D receptor Fc chimera and compared to isotype control (dotted). Cell surface expression of NKG2DL on infected cells was determined by gating for RhCMV IE2^+^ cells. The results shown are representative of two independent experiments. **D)** and **E)** Increased NK cell stimulation by ΔRh159. PBMCs were isolated from Cynomologus Macaques via Ficoll density separation, followed by sorting for NK cells (CD3^-^/CD8^+^/NKG2A^+^). NK cells were stimulated with IL-15 and IL-2 overnight followed by 4 h incubation with autologous fibroblasts infected with either RhCMV or ΔRh159 (MOI = 5) or left uninfected for 48 h. Infection was monitored by staining for IE2. MHC-I negative K562 cells were used as a positive control. Staining for CD107a, a marker for degranulation, was used to monitor NK cell activation. **D)** shows results from one representative animal. **E**) shows average results from three individual animals pooled from two individual experiments. Statistics were performed using repeated measures ANOVA with Bonferroni’s correction (*CI>95%), and bars indicate SD.

To verify that deletion of Rh159 would similarly impact the expression of RM NKG2DLs we generated telomerized RM fibroblasts (TRF) transduced with lentivectors expressing RM MIC1, RM MIC2, RM ULBP1, RM ULBP2, and RM ULBP3 (Fig 6A). Similar to the human ligands, we observed that all RM NKG2DLs were downregulated by RhCMV and that expression of RM MIC2, the equivalent of MICB, was restored upon Rh159 deletion (Fig 6A). Similar to human ULBP2, we observed an increase of RM ULBP2 whereas the MICA equivalent, MIC1, surface levels remained low in the absence of Rh159 and was only marginally increased compared to RhCMV (Fig 6A). Thus, for both human and RM NKG2DLs deletion of Rh159 predominantly increased MICB/MIC2 consistent with Rh159 being predominantly responsible for MIC2 retention whereas additional RhCMV proteins likely prevent MIC1 and ULBPs surface expression. Consistent with MIC2 upregulation, ΔRh159 also increased expression of endogenous rhesus MICs compared to RhCMV (Fig 6B). Moreover, we observed increased binding of soluble human NKG2D receptor to ΔRh159-infected versus RhCMV-infected TRF (Fig 6C). To directly determine whether increased MIC2 expression by ΔRh159 impacted NK cell stimulation, we infected primary fibroblasts derived from three individual macaques with RhCMV or ΔRh159 and monitored stimulation of purified, autologous NK cells at 48 hpi. Macaque NK cells were magnetically sorted from PBMC using antibodies to NKG2A with the majority of NKG2A+ NK cells expected to co-express NKG2D [36]. Following co-incubation with autologous fibroblasts for 4h, NK cell activation was measured by CD107a (lysosomal-associated membrane protein 1), a surrogate marker for degranulation [37]. NK cells efficiently recognized the MHC-I-deficient human cell line K562, which was used as a positive control (Fig 6D). In contrast, NK cells responded less to autologous fibroblasts infected with RhCMV when compared to uninfected K562 cells or to fibroblasts infected with ΔRh159 (Fig 6D). Differential NK cell stimulation was not due to different percentages of cells infected as shown by IE staining (Fig 6D). These observations demonstrate that Rh159 significantly limits NK cell stimulation by RhCMV-infected cells.

### Rh159 is essential for infection

**Fig 7:**
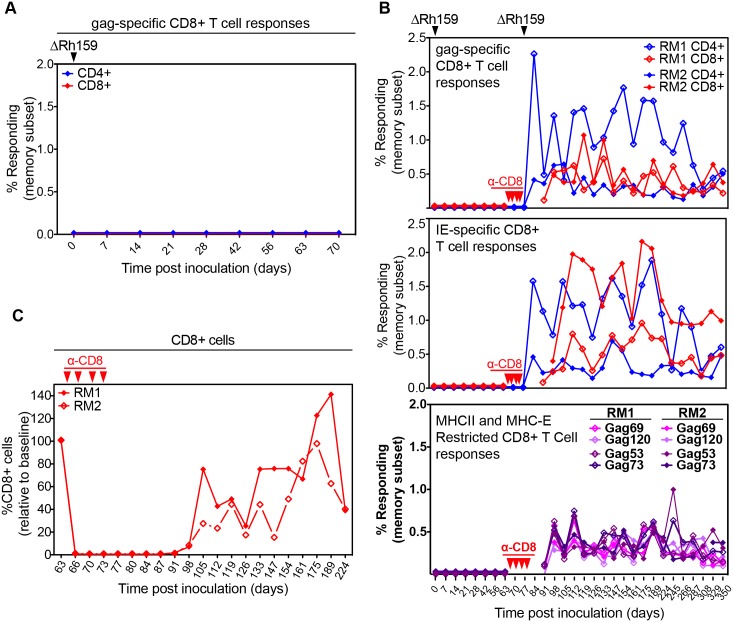
Primary infection of rhesus macaques requires evasion of NK cells by Rh159. **A)** Rh159 is required for superinfection. At day 0, a RhCMV+ RM was infected subcutaneously (s.c.) with 5x10^6^ PFU of ΔRh159. The SIVgag-specific T cell response in PBMC was monitored by ICCS for CD69, TNFα and IFNγ using overlapping (by 4AA) 15mer peptide mixes. Results are shown as a percentage of total memory CD4^+^ or CD8^+^ T cells. No responses above background were measured. **B)** Rh159 is required for primary infection. Two RhCMV-naïve animals were inoculated s.c. with 5x10^6^ PFU ΔRh159 and SIVgag- and RhCMV IE-specific T cells were monitored as in A). In addition, SIVgag-specific CD8+ T cell responses to 2 MHC-E restricted (Gag69 and Gag120) and 2 MHC-II-restricted (Gag53 and Gag73) supertope peptides were quantified by flow cytometric ICS. Starting on day 63, RM were treated with anti-CD8 antibody CM-T807 to deplete CD8^+^ cells and RM were re-inoculated with 5x10^6^ PFU of ΔRh159 on day 64. **C)** The relative frequencies of CD8^+^ small CD3- lymphocytes in whole blood (WB) of each animal were monitored during CM-T807 treatment.

To determine whether decreased NK cell evasion impedes the ability of ΔRh159 to overcome pre-existing immunity during super-infection, as we reported for T cell evasion [28], we inoculated a CMV-positive animal with ΔRh159. Since the SIVgag protein was used to replace Rh159 in this construct, we monitored the T cell responses to SIVgag as a surrogate marker for infection. As shown previously, as little as 100 PFU of RhCMV encoding SIVgag are sufficient to elicit SIVgag-specific T cell responses in RhCMV-positive animals [28]. Surprisingly, however, even at a dose of 5x10^6^ PFU, ΔRh159 did not induce SIVgag-specific T cell responses upon sub-cutaneous inoculation (Fig 7A). Since T cell responses are a sensitive measure of infection, these results suggested that Rh159 is either required to overcome pre-existing immunity (as shown previously for the MHC-I downregulating genes Rh182-189 (US2-11) [28]) or Rh159 is required for infection regardless of the immune status of the recipient. To determine whether Rh159 was required for primary infection we inoculated two CMV-naïve RM with the same dose of ΔRh159 and monitored the T cell response to SIVgag as well as to RhCMV IE. However, neither SIVgag nor IE-specific T cells were detected in either of the two CMV-naive RM at any time post-infection (Fig 7B). In contrast, wild type RhCMV never failed to induce SIV-specific T cell responses at comparable doses in hundreds of RM inoculated to date [21, 23, 25, 28]. Furthermore, SIVgag-specific T cells are observed regardless of the promoter driving gag expression, e.g. replacing Rh189, which results in SIVgag expression levels that are lower than that of Rh159 *in vitro* (S2 Fig) resulted in robust SIVgag-specific T cell responses *in vivo* [25]. These results thus suggest that RhCMV was unable to establish infection in the absence of Rh159. To determine whether increased clearance by NK cells prevented infection by ΔRh159, we wanted to monitor infection under conditions that temporarily eliminate NK cells. Since in RM all NK cells express CD8, depletion of CD8+ cells eliminates both CD8+ T cells and NK cells [38]. During the first days of infection, CMV-naïve animals lack CMV-specific CD8^+^ T cells and, for this reason, evasion of CD8+ T cells is not required for primary infection [28]. Therefore, CD8-depletion was used to temporarily eliminate NK cells and T cells on day 63 after the initial inoculation with ΔRh159 (Fig 7C). Re-inoculation of both RM with ΔRh159 resulted in SIVgag-specific and IE-specific CD4^+^ and (with some delay) CD8^+^ T cell responses (Fig 7B). These responses remain significantly above background levels to date and are likely to persist for the life of the 2 RM. Since depletion of non-specific CD8^+^ T cells was unlikely to impact infection by ΔRh159, these data strongly suggested that elimination of NK cells permitted infection by ΔRh159; and, therefore, that NK cell evasion by Rh159 is essential for primary infection.

We recently reported that RhCMV (strain 68–1) elicits unconventional CD8+ T cell responses in RM that recognize peptides presented by MHC-E and MHC-II and includes “supertopes”, i.e. peptides recognized by CD8+ T cells from MHC-disparate animals [24, 25]. To determine whether deletion of Rh159 would affect CD8+ T cell specificity we monitored the CD8+ T cell response to supertopes (presented by either MHC-E or MHC-II). Upon recovery of CD8+ T cells each of the SIVgag-derived supertope peptides stimulated CD8+ T cells from ΔRh159-infected RM suggesting that ΔRh159 maintained the unconventional T cell targeting phenotype of the parental RhCMV strain (Fig 7B).

### HCMV UL16 can substitute for Rh159 in vivo

**Fig 8:**
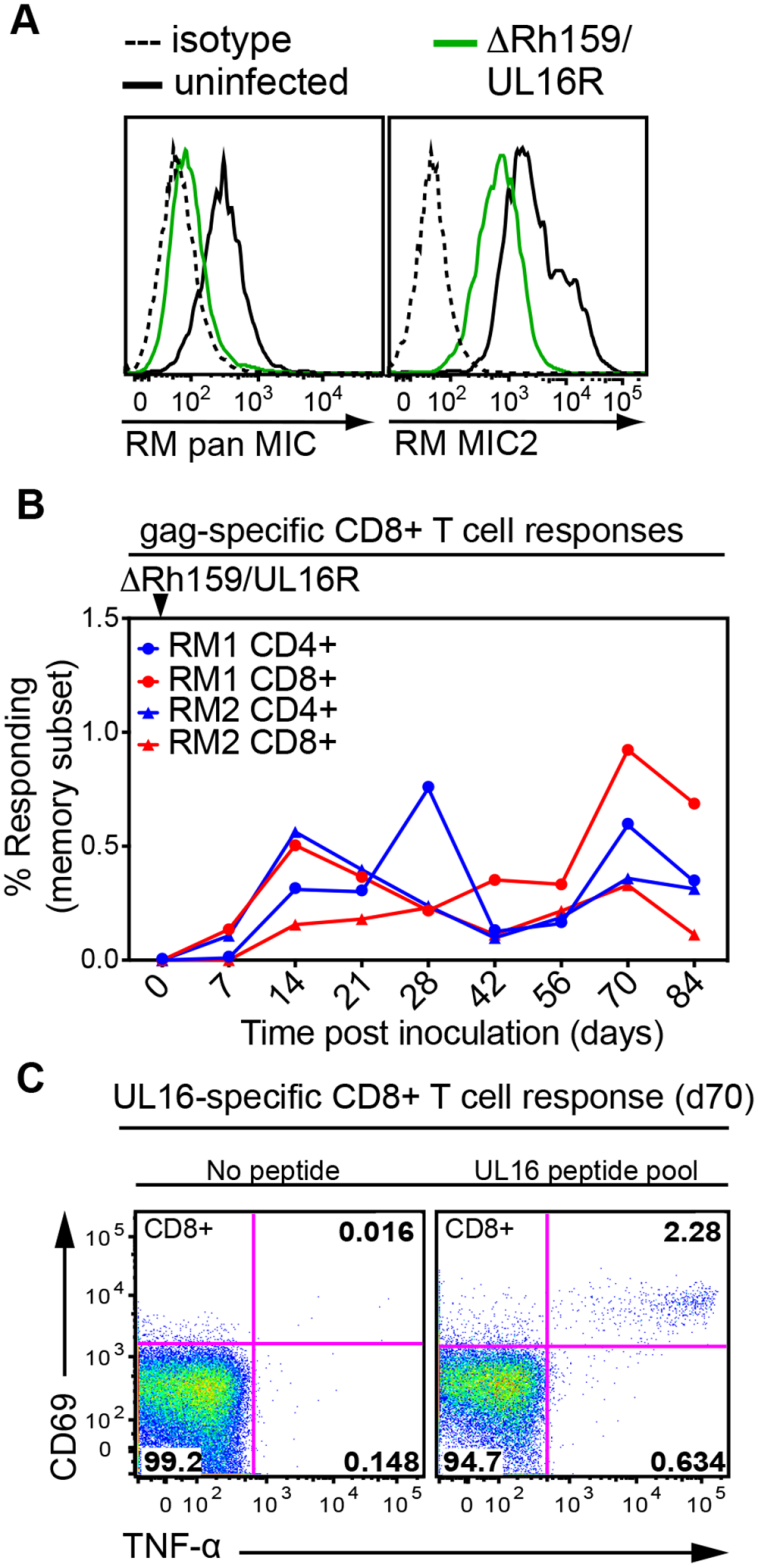
HCMV NK cell evasion protein UL16 can substitute for Rh159 during infection of rhesus macaques. **A)** Reduced surface expression of RM MICs upon infection with RhCMV expressing UL16 in place of Rh159. TRFs (left panel) or TRF-MIC2 (right panel) were infected ΔRh159/UL16R for 48h or uninfected (The corresponding ΔRh159 control is shown in Fig 6B). Endogenous MIC expression was monitored by flow cytometry with anti-RM MIC antibody whereas HA-tagged MIC2 expression was measured using anti-HA antibody. Shown is cell surface expression on RhCMV-infected cells gated for IE2^+^. The results shown are representative of at least two independent experiments. **B, C**) Superinfection of RhCMV sero+ RM by ΔRh159/UL16R. Two RhCMV sero+ RM were inoculated s.c. with 3x10^4^ PFU of ΔRh159/UL16R which contains an SIV-gag expression cassette in the Rh211 gene (S3 Fig). **B**) SIVgag-specific CD4+ and CD8+ T cell responses were monitored in PBMC by ICCS as in Fig 7 at the indicated dpi. **C**) UL16-specific T cell responses were measured on day 70 in one RM using a previously described peptide pool (UL16 peptide pool) or co-stimulation alone (no peptide) [39].

Both Rh159 and HCMV UL16 share the ability to downregulate multiple NKG2DLs, including MICB. Since UL148 does not target NKG2DLs and since RhCMV does not encode a sequence homolog of UL16, we hypothesized that, although Rh159 is the sequence homologue of UL148, it is functionally more closely related to UL16. To test this hypothesis we replaced the coding region of Rh159 with that of UL16 by BAC mutagenesis of a recombinant RhCMV that contains an SIVgag expression cassette in Rh211 [21] thus generating ΔRh159/UL16R (S3 Fig). The BAC was fully sequenced and virus recovered in RM fibroblasts expressed UL16 (S3 Fig). To determine whether the HCMV protein was able to downregulate RM NKG2DLs in the context of RhCMV, we infected RM fibroblasts with ΔRh159/UL16R and monitored the surface expression of MIC2. As shown in Fig 8A, ΔRh159/UL16R prevented the upregulation of endogenous MIC proteins and reduced surface levels of transfected MIC upon infection, whereas endogenous MIC was upregulated and transfected MIC2 was no longer downregulated in ΔRh159-infected cells compared to RhCMV (Fig 6B). To examine whether ΔRh159/UL16R would be able to infect RM despite the lack of Rh159 we inoculated two RhCMV seropositive RM with a low dose 3x10^4^ PFU of ΔRh159/UL16R and monitored the T cell response to SIVgag. Remarkably, both animals developed robust T cell responses to SIVgag suggesting that UL16 can substitute the immune evasion function of Rh159 (Fig 8B). Furthermore, using UL16-specific peptides previously used to map HCMV-ORF-specific T cell responses [39] we were able to detect a UL16-specific CD8+ T cell response when examining one of the RMs at 70dpi (Fig 8C). Although there is no sequence homology between UL16 and Rh159, the ability of both proteins to prevent the induction of NKG2DL, particularly that of MICB and MIC2, seems to promote CMV infection. These data thus further support our conclusion that preventing the induction of NKG2DL is essential for infection and strongly suggest that it is the NK cell evasion function of Rh159 that is required for infection.

## Discussion

Our data indicate that limiting NKG2DL expression on infected cells is essential for RhCMV infection. The conservation of most NKG2DLs between human and RM, together with the fact that UL16 can substitute for Rh159, renders it highly likely that HCMV will similarly depend on NK cell evasion to establish infection. Since each NKG2DL can engage NKG2D such stringent NK cell control creates an enormous selective pressure to counteract each activating ligand. Consequently, multiple NKG2DL-inhibitory gene products are expressed by HCMV, with UL16 being the quintessential NKG2DL-inhibitor acting on multiple NKG2DLs. In contrast to its host targets however, the NKG2DL-inhibitor UL16 is not conserved between HCMV and RhCMV. We identified Rh159, the homologue of HCMV UL148, as a broadly acting NKG2DL inhibitor. Similar to UL16, Rh159 is an ER-resident type I glycoprotein that retains MICA, MICB, ULBP1, and ULBP2 in the ER but not ULBP3. A tyrosine-based motif in the cytoplasmic domain of UL16 was implicated in ER-retrieval [9] whereas the luminal domain is thought to interact with NKG2DLs [11]. However, so far it has not been possible to demonstrate the formation of a stable complex between NKG2DL and UL16, or any other cytomegaloviral NKG2DL-inhibitor. In contrast, Rh159 was identified in co-immunoprecipitations with MICB consistent with a remarkably stable complex. The predicted type I topology of Rh159 suggests that its ectodomain interacts with NKG2DLs whereas the short, carboxyterminus (KRSREAH) is predicted to expose an RXR ER-retrieval motif [40] responsible for ER-retention of Rh159 and consequently NKG2DLs. However, the role of individual protein domains in Rh159 function has yet to be confirmed experimentally.

Interestingly, HCMV UL148 does not seem to interfere with NKG2DL expression despite clear homology to Rh159 suggesting that the two genes diverge functionally despite common ancestry. Homologues of UL148 are found in all primate CMVs but not in rodent CMVs, whereas UL16 and UL142 are only found in human and chimpanzee CMVs. Since the NKG2DL system predates primate evolution we speculate that the UL148 ancestor most likely evolved to counteract NKG2DL, a function that is conserved in non-human primate CMVs, but substituted with UL16 in human and ape CMVs. In HCMV, US18 and US20 complement UL16 by targeting MICA as well as UL142, which in addition targets ULBP3. Similarly Rh159 did not interfere with ULBP3 expression suggesting that additional RhCMV proteins are responsible for the reduction of ULBP3 surface expression observed in RhCMV-infected cells. Moreover, only human MICA and MICB as well as RM MIC2 were robustly upregulated in ΔRh159-infected cells suggesting that Rh159 is the only gene involved in MIC2 retention whereas Rh159 likely shares the ability to downregulate other NKG2DLs with additional RhCMV proteins. Possible candidate genes for additional MIC1 targeting are Rh199 and Rh201, the RhCMV homologs of HCMV US18 and US20 that were shown to degrade MICA via the lysosome [16]. In addition, RhCMV encodes about 20 ORFs not conserved in HCMV [26] and it is possible that some of these genes encode additional NKG2DL inhibitors. Further work will be required to identify these additional NKG2DL inhibitors and it will be interesting to determine whether upregulation of NKGDLs in their absence would lead to activation of NK cells *in vitro* and stringent control of CMV infection by NK cells *in vivo* as observed for ΔRh159.

The absolute requirement for MIC2 downregulation by Rh159 to establish infection upon sub-cutaneous inoculation was unexpected because deletion of the NKG2DL-inhibitors m138, m145, m152 or m155 from MCMV reduces viral titers, but does not prevent infection of CMV-naïve mice [2]. In fact, replacement of m152 with the murine NKG2DL Rae-1 increased CD8^+^ T cell responses to MCMV [19, 20]. This is in stark contrast to the complete absence of T cell responses observed in CMV-naïve animals inoculated with ΔRh159. We consider it unlikely that ΔRh159 established a low level infection that would not have elicited a T cell responses since CMV-specific T cell responses do not require viral dissemination or spreading from initially infected cells [41, 42]. Thus, the most likely explanation for the lack of SIVgag-specific and RhCMV IE-specific T cell responses in RM infected with ΔRh159 is that NK cells rapidly eliminated infected cells due to MIC2 induction. This conclusion is supported by the induction of SIVgag-specific and RhCMV-specific T cells by ΔRh159 upon treatment with CD8-specific antibodies or upon replacement of Rh159 with UL16, which restored MIC downregulation and the ability of ΔRh159 to infect RM. Treatment with anti-CD8 monoclonal antibody cM-T807 effectively removes both CD8^+^ T cells and NK cells from circulation [38]. However, it seems unlikely that T cell depletion permitted infection with ΔRh159, since CMV-naïve animals lack CMV-specific CD8^+^ T cells at the time of cM-T807 treatment. Moreover, we reported previously that evasion of CD8^+^ T cells is only required to establish secondary infections in CMV-immune animals, but not for primary infection [28]. Thus, although cM-T807 eliminates both T cells and NK cells, CD8^+^ NK cells were most likely responsible for the suppression of ΔRh159 infection. Interestingly, the magnitude and duration of the T cell response elicited by ΔRh159 did not diminish upon recovery of CD8^+^ cells suggesting that neither NK cells nor T cells were able to eliminate ΔRh159 once a persistent infection has been established. Similarly, we previously reported that RhCMV lacking MHC-I evasion genes infected CMV-positive animals upon CD8^+^ cell depletion and maintained a persistent infection [28]. Taken together these data suggest that both NK and T cells prevent viral spreading from the initial sites of infection, but neither innate nor adaptive cellular responses can eliminate latent viral reservoirs and clear persistent infection, even when NK or T cell immune evasion is incomplete. We thus expect to observe life long effector memory T cell responses in the anti-CD8 treated animals despite full recovery of NK and T cell responses. A possible explanation for this finding is that latent virus escapes NK and T cell clearance either by being immunologically silent or by using immune evasion mechanisms that differ from the ones used during lytic infection and viral dissemination.

In addition to inhibiting NKG2DL surface expression demonstrated here, Rh159 was previously shown to enhance infection of epithelial cells (EC) [43]. Specifically, it was demonstrated that disruption of the Rh159 gene by transposon insertion reduced growth of RhCMV (strain 68–1) in RM retinal ECs by an unknown mechanism. Thus, Rh159 seemed to mediate EC growth independently of the major determinant of EC entry, the pentameric complex gH/gL/UL128/130/131, because 68–1 lacks the UL128 and UL130 subunits [44]. Interestingly, it was reported that HCMV UL148 affects the ratio of the trimeric (gH/gL/gO) and the pentameric complex in viral particles [45]. In the absence of UL148, maturation of gH/gL/gO was impaired thus enhancing the amount of pentameric complexes and infection of ECs. Whether Rh159 similarly affects viral glycoprotein maturation is currently unknown, but it is conceivable that Rh159 might impact the maturation not only of host glycoproteins but also of viral glycoproteins. We recently reported that the spontaneous deletion of the homologs of UL128 and UL130 in RhCMV 68–1 results in the exclusive induction of unconventional CD8^+^ T cells recognizing peptides in the context of MHC-II or MHC-E [24, 25]. This unexpected function of pentameric complex components in modulating CD8^+^ T cell responses could thus potentially be further modified by Rh159. However, in CD8+-depleted animals infected with ΔRh159, the CD8+ T cell targeting phenotype was the same as that of the Rh159-intact parental strain 68–1, i.e. CD8+ T cells were restricted by MHC-E and MHC-II, but not by MHC-I, once CD8+ T cells recovered from depletion. We have not yet determined whether deletion of Rh159 would impact conventional CD8^+^ T cell responses by UL128-130-intact RhCMV. Thus, Rh159 might play multiple roles *in vivo* that range from NK cell inhibition during primary infection to affecting cell tropism to modulating adaptive T cell responses during persistent infection.

## Materials and Methods

### Cell lines and antibodies

All cell lines were cultured in Dulbecco's modified Eagle medium (DMEM) supplemented with 10% fetal bovine serum (FBS), 100 IU of penicillin/ml and 100 μg of streptomycin/ml and incubated at 37C and 5% CO_2_ except where noted. K562 cells were obtained from ATCC. 293A cells were obtained from Invitrogen. 293-CRE expressing cells were a kind gift from Ashley Moses (Oregon Health and Science University). Telomerized RM fibroblasts (TRFs), primary cynomologus fibroblasts and NK cells were obtained from animals housed at Oregon National Primate Research Center. U373-NKG2DLs were previously described [31]. TRF-RM NKG2DLs were generated using the pLVX lentivector system (Clontech) by cotransfecting pLVX, along with vectors encoding vesicular stomatitis virus G (VSV-G) (pMD2.G; Addgene 12259) and Gag/Pol (psPAX2; Addgene 12260), into TRF cells using Lipofectamine LTX (Life Technologies) according to the manufacturer’s protocol. Supernatant containing lentivirus was harvested from the transfected cells 48 h posttransfection, and used to transduce TRF-RM NKG2DLs in the presence of 5 μg/ml Polybrene (hexadimethrine bromide; Sigma-Aldrich). This process was repeated 24h later, and the resulting cell lines were grown in the presence of 3 μg/ml puromycin to select for cells that expressed the viral genes. The RM NKG2DLs were synthesized (GenScript) based upon the following accession numbers: AF055387 (MIC1), AF055388 (MIC2), XM_001082270.2 (RM ULBP1), XM_001082656.1 (RM ULBP2), and XM_001083203.2 (RM ULBP3; since the deposited RM sequence lacked a stop codon we inserted nine nucleotides (5’ GGCAGATGA 3’) at the appropriate position based on their conservation in multiple non-human primate ULBP3 sequences). Synthetic genes were cloned into pcDNA3.1(-), and subcloned into pLVX containing an eF1α promotor. All constructs were synthesized to encode an HA epitope tag on the amino terminal end, after the predicted signal sequence (SignalP 4.1).

The following primary antibodies were used for immunoprecipitation, flow cytometric and/or immunoblot analysis of U373-NKG2DLs: mouse anti-MICA (AMO1), -MICB (BMO2), -ULBP1 (AUMO2), -ULBP2 (BUMO1), and -ULBP3 (CUMO3) (Axxora), -TfR (M-A712, BD Pharmingen), -GAPDH (6C5), -CD44 (DF1485) and -V5 (E10) (Santa Cruz), -RM MIC (a kind gift from Thomas Spies[46]), -FLAG (M2) and polyclonal rabbit anti-FLAG (Sigma), and polyclonal goat anti-ULBP3 (AF1517), -ULBP2 (AF1298) and -ULBP1 (AF1380) (R&D systems). For isotype controls, mouse IgG2_a_ (02–6200) or IgG1 (MG100) (Life Technologies) were used. Chicken anti-mouse Alexa Fluor 647 (Life Technologies) and streptavidin PE-Cy7 (eBioscience) were used as secondary antibodies in FACs experiments. Goat anti-mouse IgG-HRP and donkey anti-goat IgG-HRP (Santa Cruz) were used for immunoblotting. Total surface NKG2DLs in TRFs were measured using human NKD2D-Fc chimera (R&D Systems) followed by anti-human IgG-PE (eBioscience). Human Fc-G1 (BioX-Cell) was used as isotype control. Streptavidin-APC (eBioscience) was used for intracellular secondary staining. For the NK activation assay NKG2A-PE (Z199, Beckman-Coulter) and anti-PE beads (Miltenyi Biotech) were used to magnetically sort NKG2A^+^ cells from PBMC, and subsequently stained after co-culture with autologous fibroblasts using anti-CD107a-FITC (H4A3), CD8-APC-Cy7 (SK1), IFN-γ-APC (B27) (BD Biosciences), yellow Live/Dead fixable stain (Invitrogen) and NKG2A-PE.

### Recombinant viruses

RhCMVΔRh159gag (ΔRh159) was created by BAC-recombineering [47] replacing Rh159 with an expression cassette for SIVgag in RhCMV 68–1 [48]. Briefly, primers containing 50 bp homology to regions flanking Rh159 (forward primer 5’GGTCGTTTGGTTGTTTCTCACCTATTGCTTGGTACTCTAGCT TCAGTAAG3′ and reverse primer 5’TAGTTTATAAACACACAATCACGTGGTGGT ACTGTGAACCCGCGTCGGTA-3′) were used to amplify SIV mac239 gag and a kanamycin resistance (KanR) cassette flanked by FRT sites from plasmid pCP015 and electroporated into EL250 bacteria containing the RhCMV 68–1 BAC for in vivo recombination. Upon removal of the KanR gene by FLP recombination, recombinant virus was reconstituted in primary RM fibroblasts. Self-excision of the loxP-flanked BAC-cassette in the resulting virus was confirmed by full genome sequencing.

In a two step process RhCMV ΔRh159/UL16R was generated by en passant mutagenesis [49] to precisely replace the RhCMV Rh159 coding region with that of HCMV UL16. In the first step we inserted a DNA fragment containing an I-SceI restriction site and a Kanamycin resistance cassette into UL16 of HCMV strain TR BAC [50] by homologous recombination using the following flanking primers:

Forward primer 5’*CCAGCCGCATGGTCACTAATCTTACCGTGGGCCGTTATGACTGTTTACGC* **TGCGAGAACGGTACGATGAAAATAATCGAGCGCCTCCACGTCCGATTGGG** TAGGGATAACAGGGTAATAAG-3’

Reverse primer 5’*CGGAGGGGTGTTTGGCGAGCCCGGATCCGGGCGGTCTCGGATATAGCGAG* **CCCAATCGGACGTGGAGGCGCTCGATTATTTTCATCGTACCGTTCTCGCA** AGAGCGCTTTTGAAGCTGG-3’.

Next, we amplified the UL16 gene containing the I-SceI site and Kanamycin resistance cassette by PCR with primers containing 50 bp homology sequences flanking Rh159:

Forward primer: 5’*GGTCGTTTGGTTGTTTCTCACCTATTGCTTGGTACTCTAGCTTCAGTAAG* ATGGAGCGTCGCCGAGGTACG-3’

Reverse primer: 5’*GTTTATAAACACACAATCACGTGGTGGTACTGTGAACCCGCGTCGGTATC* AGTCCTCGGTGCGTAACC3-’).

In the second step, the PCR fragment was transformed into E.coli strain GS1783 [49] harboring RhCMV 68-1/gag and Rh159 was replaced with the UL16/I-SceI/KanR fragment by homologous recombination and Kanamycin selection. Next, we removed the I-SceI/KanR cassette by inducing red recombinases and the I-SceI restriction enzyme. Cleavage of the BAC-DNA by the I-SceI enzyme followed by red-dependent recombination between two homologous sequences flanking the I-SceI/KanR fragment (shown in bold in above primer set) results in the precise excision of the I-SceI/KanR cassette and the restoration of the intact HCMV UL16 ORF. The resulting ΔRh159/UL16R BAC was characterized by next generation sequencing of the entire BAC.

To generate AdRh159FL, Rh159 was amplified from viral DNA isolated from TRFs infected with RhCMV using the following primers: 5' CGGGATCCCGCCACCATGGCCTACAACAG-3' and 5’GGAATTCCTTACTTATCGTCGTCATCCTTGTAGTCATGAGCTTCACGACTGCGTT-3’. The PCR fragment was cloned into pADTet7, and transfected into 293-CRE cells with psi5 helper virus [51]. RhCMVΔRh189gag and RhCMV68-1/gag were previously described [25]. Adenovirus expressing UL148 and empty vector control were a kind gift from Richard Stanton (Cardiff University) [52].

### Immunoblotting and pulse-chase experiments

U373-NKG2DL cells were lysed in PBS 1%NP-40 and HALT protease inhibitor (Thermo Fisher) followed by SDS-PAGE protein separation and transfer to PVDF. Immunoblotted proteins were detected with ECL2 (Thermo Scientific). For Pulse-chase experiments, U373-NKG2DL cells were starved for 1 h in DMEM minus cysteine (cys) and methionine (met) supplemented with 10% FBS (pulse media). After starve period, EXPRE^35^S^35^S Protein Labeling Mix (PerkinElmer) was added to pulse media at 150 μCi/10^6^ cells for 30 min. Cells were chased for the indicated times then lysed, digested, and immunoprecipitates were separated by SDS-PAGE and detected by autoradiography. Digestions using EndoH (Roche) or PNGaseF (NEB) were performed according to manufacturer’s instructions.

### Flow cytometric analysis

Cells were harvested, washed in 3% FBS/PBS, incubated with primary antibody followed by fluorophore-conjugated secondary antibody. Cells were then fixed in 1% paraformaldehyde, permeabilized in 0.1% triton-X100/PBS, washed and incubated with biotinylated anti-IE2 antibody (DBX biotin labeling kit, Molecular probes) followed by fluorophore-conjugated streptavidin. Flow cytometry data were acquired on LSR II (BD Biosciences) and analyzed with FlowJo X (v.10.0.7, Tree Star).

### NK activation assays

NKG2A^+^ cells sorted from PBMC were plated overnight in RPMI-1640, 15% FBS, 100 IU/ml of IL-2, and 10 ng/ml of IL-15 (NK media). NKG2A^+^ cells (2.5 x 10^6^/ml) were incubated with autologous fibroblasts (1.25 x 10^5^ IE-2 expressing cells/ml) for 30 min at 37°C in the presence of anti-CD107/FITC. Brefeldin A (10 μg/ml) and GolgiStop (1 μl/ml; BD Biosciences) were added after 30 min and samples were incubated at 37°C for 8 hrs. Cells were surface stained, fixed with 2% paraformaldehyde, and then permeabilized for intracellular IFN-γ staining with 1x PBS containing 10% FBS and saponin (1 g/L).

### Mass spectrometry

Proteins excised from Coomassie gels were trypsinized and peptides were analyzed by LC/MS-MS using an LTQ Velos Pro linear ion trap (Thermo Scientific) to collect data-dependent MS/MS data [53]. Sequest (version 28, revision 12) was used to search MS2 Spectra against a March 2012 version of the Sprot human FASTA protein database, with added sequences from the Uniprot Rhesus Cytomegalovirus and concatenated sequence-reversed entries to estimate error thresholds and 179 common contaminant sequences and their reversed forms. Database processing was performed with python scripts (ProteomicAnalysisWorkbench.com). SEQUEST results were filtered to strict peptide and protein false discovery rates (FDRs), estimated from the number of matches to sequence-reversed peptides, using PAW software [54]. Independent FDR control was performed, resulting in a 4.3% FDR for protein discovery (43 identifications to forward sequence proteins and 2 to reverse-sequenced proteins). 9 MS/MS spectra assigned to forward sequence peptides and none to reverse-sequence peptides from any entry in the database.

### Animal studies

Five male, purpose-bred, Indian-origin RM (*Macaca mulatta*) were used: three animals being RhCMV-positive and two were specific-pathogen free, including RhCMV. ΔRh159 or ΔRh159/UL16R was inoculated subcutaneously at 5 × 10^6^ PFU or 3 x 10^4^ PFU, respectively. For CD8^+^ cell depletion, RM were treated with 10, 5, 5 and 5 mg/kg of the anti CD8 mab cM-T807 one day prior to inoculation with 5 × 10^6^ PFU ΔRh159 and on days 2, 6, and 9 post inoculation, respectively. Antigen-specific CD4^+^ and CD8^+^ T cell responses were measured in mononuclear cell preparations from blood by ICCS [21]. Briefly, sequential 15-mer peptides (overlapping by 11 amino acids) comprising SIV_MAC239_gag or RhCMV IE or four core (optimal) SIVgag supertope peptides (2 each MHC-E- and MHC-II-restricted) [25]) were used in the presence of co-stimulatory CD28 and CD49d monoclonal antibodies (BD Biosciences). To detect UL16-specific responses we used a previously described overlapping peptide pool [39]. Cells were incubated with pooled peptides and co-stimulatory molecules for 1h, followed by addition of Brefeldin A (Sigma-Aldrich) for an additional 8h. Co-stimulation without antigen served as a background control. Cells were then stained with fluorochrome-conjugated monoclonal antibodies, flow cytometric data collected on a LSR II (BD Biosciences) and data analyzed using the FlowJo software program (version 8.8.7; Tree Star). Responses frequencies (CD69^+^/TNFα^+^ and/or CD69^+^/IFNγ^+)^ were first determined in the overall CD4^+^ and CD8^+^ T cell population and then memory corrected (with memory T cell subset populations delineated on the basis of CD28 and CD95 expression).

### Ethics statement

IACUC Project Number 0691, Protocol number IS00002845, and started 05/01/09 The Five purpose-bred male RM (Macaca mulatta) of Indian genetic background were used with consent of the Oregon National Primate Research Center Animal Care and Use Committee, under the standards of the US National Institutes of Health Guide for the Care and Use of Laboratory Animals.

## Supporting Information

S1 Fig Alignments of human and rhesus NKG2DLs. Pairwise alignment of human MICA (AAD52060), MICB (AAB71643), Hu ULBP1 (AF304377), Hu ULBP2 (AF304378), Hu ULBP3 (AF304378) and RM MIC1 (AAC67495.1), RM MIC2 (AAC67496.1), RM ULBP1 (XP_001082270), RM ULBP2 (XP_001082656), and RM ULBP3 (XP_001083203). The amino acid sequences were compared after the predicted signal sequence (PSI/TM-Coffee [1]) had been removed. Intensity of purple shading indicates level of sequence conservation (Jalview 2, [2]).
(TIF)

S2 Fig Characterization of RhCMVΔRh159. A) Deletion of Rh159 was confirmed by RT-PCR. RM fibroblasts were infected with either RhCMV 68–1 (WT) or ΔRh159 at an MOI of 3 for 48 h. RNA was isolated and used for RT-PCR using primers specific for Rh159, Rh160 and GAPDH. B) Confirmation of SIVgag expression. RM fibroblasts were non-infected (NI) or infected as in A with RhCMV ΔRh189 [3] or with ΔRh159. In both constructs, the viral gene was deleted by replacing the ORF with FLAG-tagged SIVgag. Cells were lysed in1%NP40 and immunoblotted with mAbs for FLAG, RhCMV IE2 or cellular GAPDH as loading control. C) In vitro growth of ΔRh159 in rhesus and human cells. TRFs or U373s were infected with either RhCMV 68–1 (WT) or ΔRh159 at an MOI = 0.1 or MOI = 3 for multistep or single step growth curves, respectively. Virus titer in the supernatant was determined by TCID_50_ on the days indicated. D) Sequencing coverage map for RhCMVΔRh159. Upon Next Generation Sequencing of ΔRh159, all sequencing reads passing quality control were aligned to the *de novo* assembled consensus sequence of the viral genome. Top: Sequence coverage is graphically depicted as number of reads per nucleotide position. Bottom: ORF map of the consensus sequence. The SIVgag sequence replacing the Rh159 ORF is highlighted as well as the loxP site remaining after Cre-mediated excision of the BAC cassette after reconstitution of virus in fibroblasts. “TR” indicates terminal repeat sequences. E) Genome alignment of RhCMVΔRh159 with the parental WT (BAC-derived RhCMV 68–1 virus). The bar indicates the percentage of nucleotide identity between both virus sequences with green being 100% identical. The only sequence difference between the parental virus and RhCMVΔRh159 represents the location of the replacement of Rh159 with SIVgag indicating that no unwanted recombinations or spurious mutations are present in the majority sequence.
(TIF)

S3 Fig Characterization of ΔRh159/UL16R. A) Replacement of Rh159 with UL16 was confirmed by RT-PCR. RM fibroblasts were infected with either RhCMV 68–1 (WT) or ΔRh159/UL16R at an MOI of 3. At 48 hpi, total RNA was isolated from cell lysates and RT-PCR was performed using primers specific for Rh159, Rh160 and GAPDH. Additionally, UL16 expression was confirmed by RT-PCR using RNA isolated from RM fibroblasts infected with ΔRh159/UL16R. To confirm UL16 primer specificity we used HCMV-TR BAC DNA for control C. B) Confirmation of SIVgag expression. RM fibroblasts were uninfected or infected as in A with ΔRh159 or ΔRh159/UL16R lysed in 1% NP40 and immunoblotted with mAbs for IE2, FLAG and GAPDH. C) Confirmation of UL16 expression. Fibroblasts were infected as in A with the indicated viruses. Upon lysis, cell lysates were treated with PNGase where indicated prior to SDS-PAGE and immunoblotting with anti-UL16 antibodies. The position of glycosylated (UL16) or deglycosylated UL16 (S) is indicated. All other bands are non-specific. D) Sequencing coverage map for RhCMVΔRh159/UL16R. Upon Next Generation Sequencing of RhCMVΔRh159/UL16R BAC DNA all sequencing reads passing quality control were aligned to the *de novo* assembled consensus sequence. Top: Sequence coverage is depicted as number of reads per nucleotide position. Bottom: ORF map of the consensus genome sequence with the UL16 ORF (replacing the Rh159 ORF) highlighted as well the BAC cassette and the SIVgag-expression cassette inserted into ORF Rh211. E) Alignment of the RhCMVΔRh159/UL16R BAC consensus sequence with the parental RhCMV 68–1 BAC. The bar indicates the percentage of nucleotide identity between both BAC sequences with green being 100% identical. Importantly, the only sequence mismatches were detected at the genome locations corresponding to Rh159 that was replaced with UL16 and Rh211 in which the SIVgag expression cassette had been inserted (black arrows). This demonstrates that no other genome regions were inadvertently affected during the construction of RhCMVΔRh159/UL16R.
(TIF)

S1 Text Supplemental Materials and Methods. (DOCX)
