# Supplementary figures and images for: Natural Killer Cell Evasion Is Essential for Infection by Rhesus Cytomegalovirus
Source: PLoS Pathog. 2016 Aug 31;12(8):e1005868. doi: 10.1371/journal.ppat.1005868 (PMC5006984; doi:10.1371/journal.ppat.1005868)

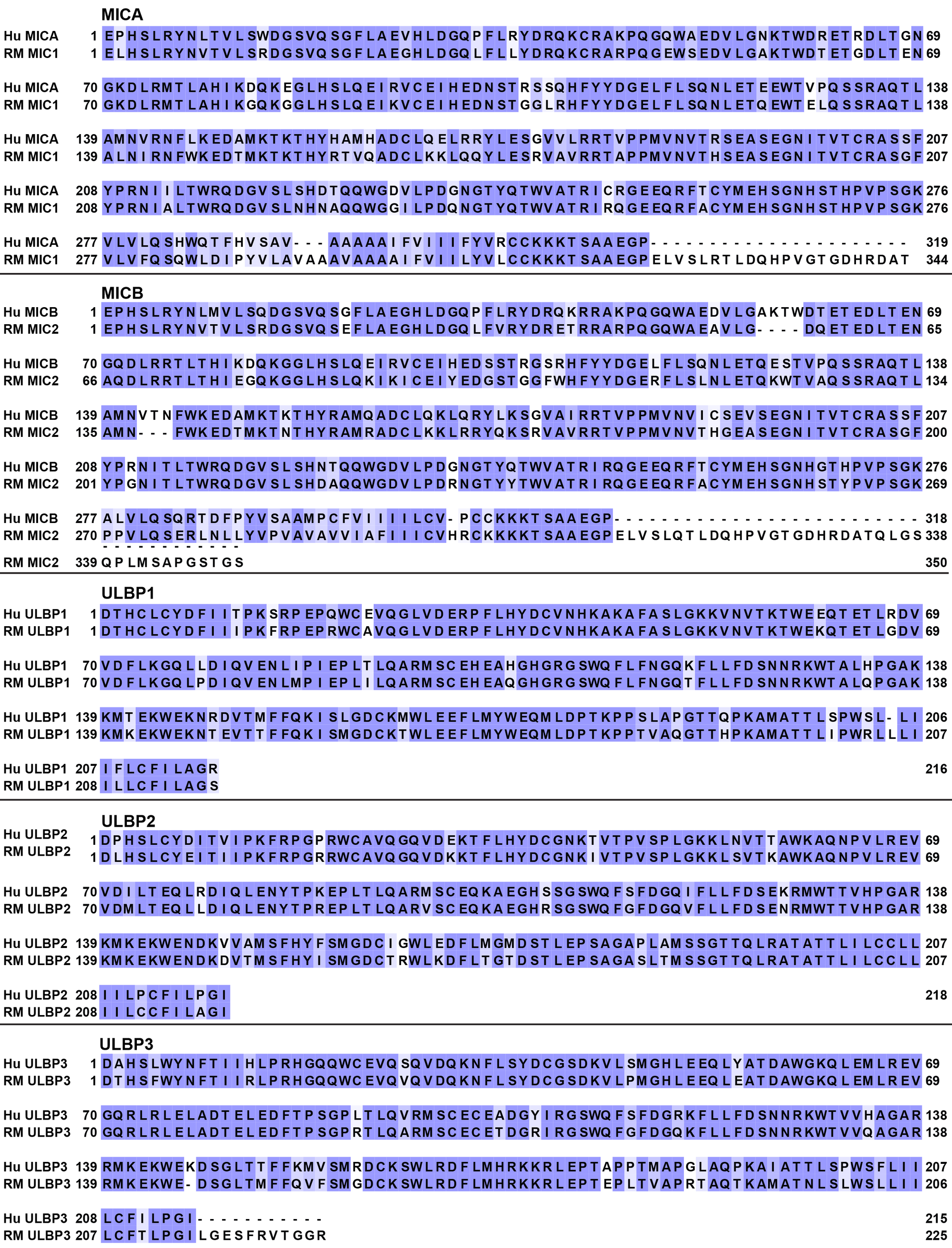

Supplement: S1 Fig — Pairwise alignment of human MICA (AAD52060), MICB (AAB71643), Hu ULBP1 (AF304377), Hu ULBP2 (AF304378), Hu ULBP3 (AF304378) and RM MIC1 (AAC67495.1), RM MIC2 (AAC67496.1), RM ULBP1 (XP_001082270), RM ULBP2 (XP_001082656), and RM ULBP3 (XP_001083203). The amino acid sequences were compared after the predicted signal sequence (PSI/TM-Coffee [1]) had been removed. Intensity of purple shading indicates level of sequence conservation (Jalview 2, [2]). (TIF) [file ppat.1005868.s001.tif]

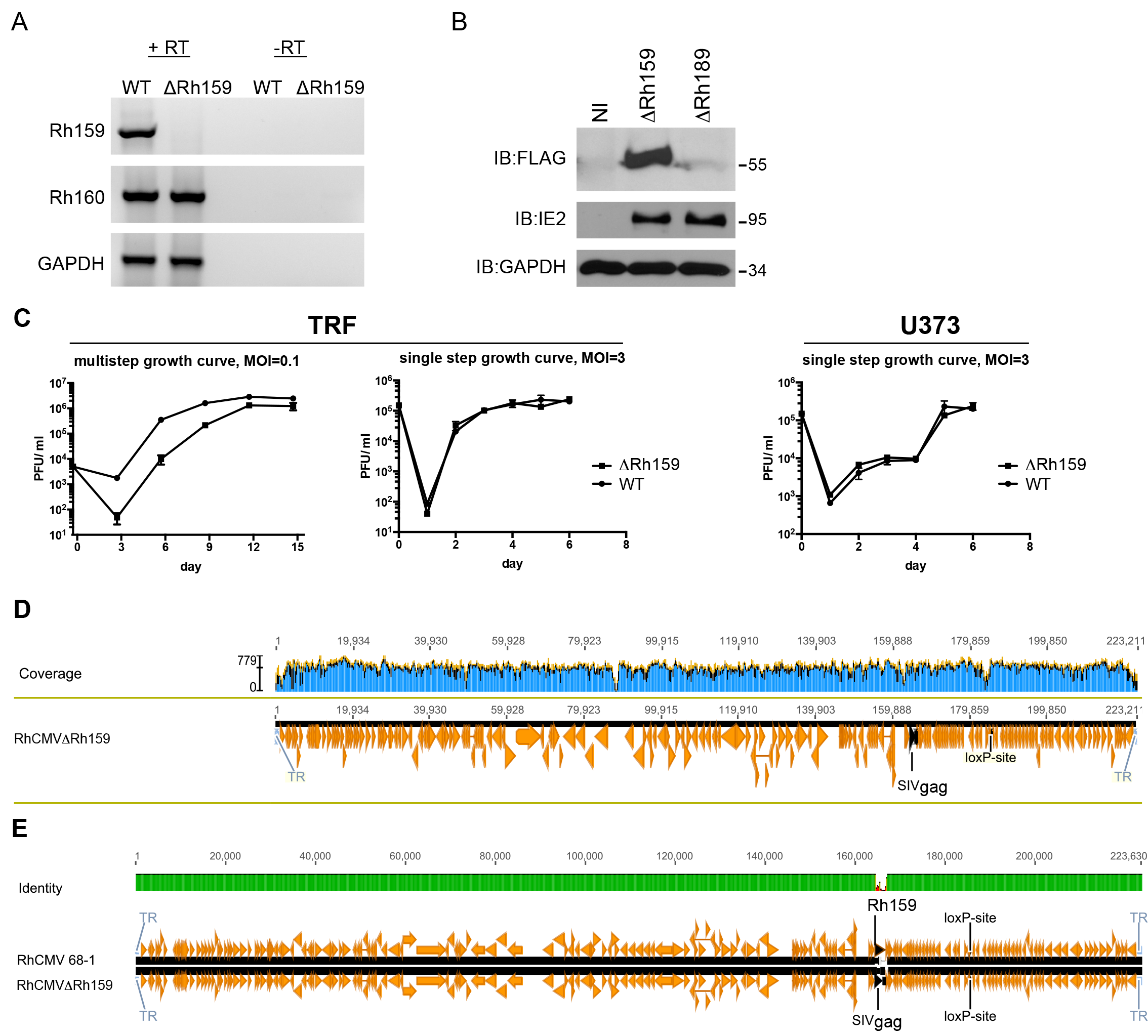

Supplement: S2 Fig — A) Deletion of Rh159 was confirmed by RT-PCR. RM fibroblasts were infected with either RhCMV 68–1 (WT) or ΔRh159 at an MOI of 3 for 48 h. RNA was isolated and used for RT-PCR using primers specific for Rh159, Rh160 and GAPDH. B) Confirmation of SIVgag expression. RM fibroblasts were non-infected (NI) or infected as in A with RhCMV ΔRh189 [3] or with ΔRh159. In both constructs, the viral gene was deleted by replacing the ORF with FLAG-tagged SIVgag. Cells were lysed in1%NP40 and immunoblotted with mAbs for FLAG, RhCMV IE2 or cellular GAPDH as loading control. C) In vitro growth of ΔRh159 in rhesus and human cells. TRFs or U373s were infected with either RhCMV 68–1 (WT) or ΔRh159 at an MOI = 0.1 or MOI = 3 for multistep or single step growth curves, respectively. Virus titer in the supernatant was determined by TCID50 on the days indicated. D) Sequencing coverage map for RhCMVΔRh159. Upon Next Generation Sequencing of ΔRh159, all sequencing reads passing quality control were aligned to the de novo assembled consensus sequence of the viral genome. Top: Sequence coverage is graphically depicted as number of reads per nucleotide position. Bottom: ORF map of the consensus sequence. The SIVgag sequence replacing the Rh159 ORF is highlighted as well as the loxP site remaining after Cre-mediated excision of the BAC cassette after reconstitution of virus in fibroblasts. “TR” indicates terminal repeat sequences. E) Genome alignment of RhCMVΔRh159 with the parental WT (BAC-derived RhCMV 68–1 virus). The bar indicates the percentage of nucleotide identity between both virus sequences with green being 100% identical. The only sequence difference between the parental virus and RhCMVΔRh159 represents the location of the replacement of Rh159 with SIVgag indicating that no unwanted recombinations or spurious mutations are present in the majority sequence. (TIF) [file ppat.1005868.s002.tif]

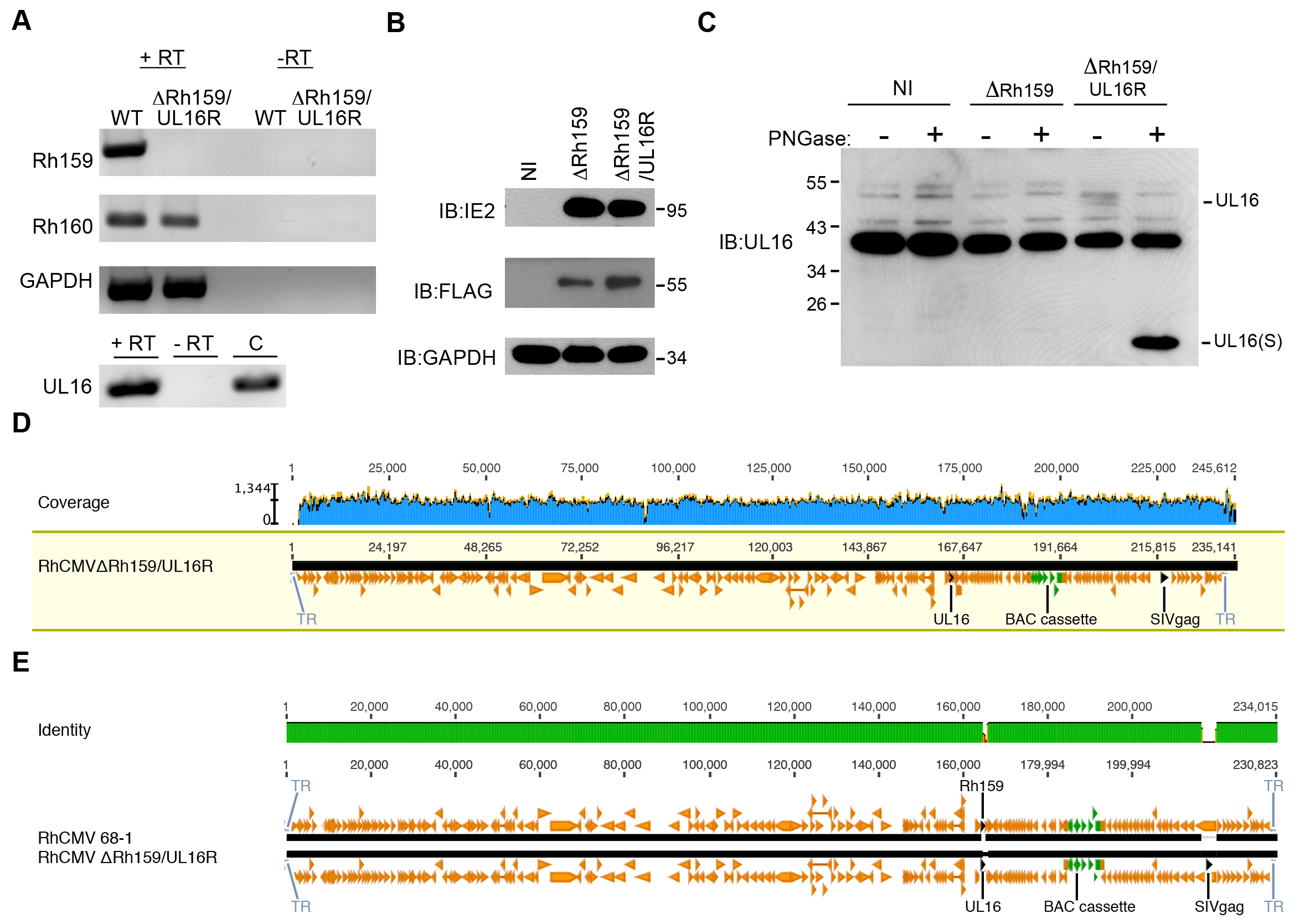

Supplement: S3 Fig — A) Replacement of Rh159 with UL16 was confirmed by RT-PCR. RM fibroblasts were infected with either RhCMV 68–1 (WT) or ΔRh159/UL16R at an MOI of 3. At 48 hpi, total RNA was isolated from cell lysates and RT-PCR was performed using primers specific for Rh159, Rh160 and GAPDH. Additionally, UL16 expression was confirmed by RT-PCR using RNA isolated from RM fibroblasts infected with ΔRh159/UL16R. To confirm UL16 primer specificity we used HCMV-TR BAC DNA for control C. B) Confirmation of SIVgag expression. RM fibroblasts were uninfected or infected as in A with ΔRh159 or ΔRh159/UL16R lysed in 1% NP40 and immunoblotted with mAbs for IE2, FLAG and GAPDH. C) Confirmation of UL16 expression. Fibroblasts were infected as in A with the indicated viruses. Upon lysis, cell lysates were treated with PNGase where indicated prior to SDS-PAGE and immunoblotting with anti-UL16 antibodies. The position of glycosylated (UL16) or deglycosylated UL16 (S) is indicated. All other bands are non-specific. D) Sequencing coverage map for RhCMVΔRh159/UL16R. Upon Next Generation Sequencing of RhCMVΔRh159/UL16R BAC DNA all sequencing reads passing quality control were aligned to the de novo assembled consensus sequence. Top: Sequence coverage is depicted as number of reads per nucleotide position. Bottom: ORF map of the consensus genome sequence with the UL16 ORF (replacing the Rh159 ORF) highlighted as well the BAC cassette and the SIVgag-expression cassette inserted into ORF Rh211. E) Alignment of the RhCMVΔRh159/UL16R BAC consensus sequence with the parental RhCMV 68–1 BAC. The bar indicates the percentage of nucleotide identity between both BAC sequences with green being 100% identical. Importantly, the only sequence mismatches were detected at the genome locations corresponding to Rh159 that was replaced with UL16 and Rh211 in which the SIVgag expression cassette had been inserted (black arrows). This demonstrates that no other genome regions were inadvertently affected during the construc [file ppat.1005868.s003.tif]
