# Supplementary material for: Natural Killer Cell Evasion Is Essential for Infection by Rhesus Cytomegalovirus
Source: PLoS Pathog. 2016 Aug 31;12(8):e1005868. doi: 10.1371/journal.ppat.1005868 (PMC5006984; doi:10.1371/journal.ppat.1005868)
Supplement: S1 Text — (DOCX) [file ppat.1005868.s004.docx]

**S1 text. Supplemental Materials and Methods.**

**RT-PCR**

Total RNA was extracted and treated with DNase using the NucleoSpin RNA isolation kit (Machery Nagel) according to the manufacturer's protocol. The concentration of the RNA samples was measured using the NanoDrop 1000 Spectrophotometer (Thermo Scientific). Single-stranded cDNA was made from total RNA using random hexamers (TaKaRa) to prime first-strand synthesis by Maxima Reverse Transcriptase (Thermo Scientific) as recommended by the manufacturer. RT-PCR of cDNA was performed using Platinum Taq DNA Polymerase (Invitrogen) in conjunction with the following primer sets to detect mRNA expression:

Rh159 Fw: 5’CCTAGAGCTTACATCTTATCTC-3’

Rh159 Rev: 5’CCAACAGTGGTGAAATTCAAGG-3’

Rh160 Fw: 5’CGCCACCTCGAATATCACGA -3’

Rh160 Rev: 5’GGACGAGCGATGTCCGTATT -3’

GAPDH Fw: 5’CAGAAGACTGTGGATGGCCC -3’

GAPDH Rev: 5’GTCAAAGGTGGAGGAGTGGG -3’

UL16 Fw: 5’CCGCAATTTCAGCGACATC-3’

UL16 Rev: 5’ACCAACACGATGTCTCTCG-3’.

HCMV TR strain BAC DNA was used a positive control for UL16 primers.

**Supplemental references**

1. Chang JM, Di Tommaso P, Taly JF, Notredame C. Accurate multiple sequence alignment of transmembrane proteins with PSI-Coffee. BMC bioinformatics. 2012;13 Suppl 4:S1.

2. Waterhouse AM, Procter JB, Martin DM, Clamp M, Barton GJ. Jalview Version 2--a multiple sequence alignment editor and analysis workbench. Bioinformatics (Oxford, England). 2009;25(9):1189-91.

3. Hansen SG, Sacha JB, Hughes CM, Ford JC, Burwitz BJ, Scholz I, et al. Cytomegalovirus vectors violate CD8+ T cell epitope recognition paradigms. Science. 2013;340(6135):1237874.
